# Determinants of the Inhibition of DprE1 and CYP2C9 by Antitubercular Thiophenes

**DOI:** 10.1002/anie.201707324

**Published:** 2017-09-07

**Authors:** Renhe Liu, Xiaoxuan Lyu, Sarah M Batt, Mei‐Hui Hsu, Michael B Harbut, Catherine Vilchèze, Bo Cheng, Kehinde Ajayi, Baiyuan Yang, Yun Yang, Hui Guo, Changyou Lin, Fei Gan, Chen Wang, Scott G. Franzblau, William R. Jacobs, Gurdyal S. Besra, Eric F. Johnson, Mike Petrassi, Arnab K. Chatterjee, Klaus Fütterer, Feng Wang

**Affiliations:** ^1^ California Institute for Biomedical Research (Calibr) La Jolla CA 92037 USA; ^2^ School of Biosciences University of Birmingham Birmingham B15 2TT UK; ^3^ Department of Molecular Medicine The Scripps Research Institute La Jolla CA 92037 USA; ^4^ Howard Hughes Medical Institute Department of Microbiology and Immunology Albert Einstein College of Medicine Bronx NY 10461 UK; ^5^ Institute for Tuberculosis Research College of Pharmacy University of Illinois Chicago IL 60612 USA

**Keywords:** anti-tubercular drugs, CYP2C9, DprE1, drug development, drug–drug interactions

## Abstract

Mycobacterium tuberculosis (Mtb) DprE1, an essential isomerase for the biosynthesis of the mycobacterial cell wall, is a validated target for tuberculosis (TB) drug development. Here we report the X‐ray crystal structures of DprE1 and the DprE1 resistant mutant (Y314C) in complexes with TCA1 derivatives to elucidate the molecular basis of their inhibitory activities and an unconventional resistance mechanism, which enabled us to optimize the potency of the analogs. The selected lead compound showed excellent in vitro and in vivo activities, and low risk of toxicity profile except for the inhibition of CYP2C9. A crystal structure of CYP2C9 in complex with a TCA1 analog revealed the similar interaction patterns to the DprE1–TCA1 complex. Guided by the structures, an optimized molecule was generated with differential inhibitory activities against DprE1 and CYP2C9, which provides insights for development of a clinical candidate to treat TB.

*Mtb* Decaprenylphosphoryl‐β‐d‐ribofuranose 2‐oxidase (DprE1) is a flavin adenine dinucleotide (FAD)‐dependent enzyme, which together with decaprenylphosphoryl‐d‐2‐ketoerythropentose reductase (DprE2), converts decaprenylphosphoryl‐beta‐d‐ribose (DPR) to decaprenylphosphoryl‐beta‐d‐arabinofuranose (DPA), an essential cell wall component.^1^ Conditional knockdown studies showed that loss of *dprE1* results in a strong bactericidal effect in vitro.^2^ Multiple covalent^3^ and non‐covalent^4^ DprE1 inhibitors have been identified and showed in vitro and in vivo activities against *Mtb*, further validating DprE1 as an attractive anti‐tuberculosis (TB) drug target.^5^

We previously reported the identification of TCA1 from a cell‐based phenotypic screen, and demonstrated it is a DprE1 inhibitor.^6^ Since then we have generated new derivatives of TCA1 to improve its in vitro potency, PK properties, and in vivo efficacy. When analyzing toxicity profiles, we noticed that many TCA1 analogs show inhibition of CYP2C9, one of six major cytochrome P450 enzymes which determine the clearance of 75 % of marketed drugs. Therefore, inhibition of P450 enzymes such as CYP2C9 can potentially lead to drug–drug interactions, which may cause serious issues for combinatory drug regimen for TB treatment.^7^

Here we report the X‐ray crystal structures of WT DprE1, a TCA1‐resistant DprE1 mutant, and CYP2C9 in complex with TCA1 analogs. Along with structure activity relationship studies, the mechanism of resistance by the mutant DprE1 enzyme and the tight correlation between DprE1 and CYP2C9 inhibition was elucidated. We further generated analogs with excellent in vitro and in vivo activities against *Mtb*, and selectively targeting DprE1 over CYP2C9.

In the DprE1–TCA1 structure, TCA1 showed a planar conformation and tight fit in the DprE1 active site. The benzothiazole core is oriented parallel to the FAD isoalloxazine ring and the thiophene carboxamide resides in small hydrophobic pocket. Besides substantial hydrophobic interactions, multiple hydrogen bonding interactions are formed between TCA1 and residues Lys418, His132, and Ser228 (Figure S1 in the Supporting Information, SI). Guided by the structure, our medicinal chemistry effort focused on the benzothiazole core (red), thiophene carboxamide (green) and the acylcarbamate (blue) of TCA1 (Figure 1).

**Figure 1:**
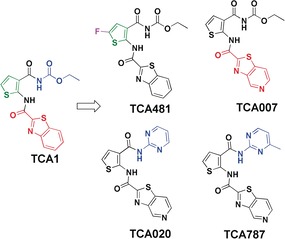
SAR optimization strategy for TCA1 analogs. Benzothiazole core (red), thiophene carboxamide (green) and acylcarbamate (blue).

The Gln334 amide group and the Tyr60 hydroxy group are only 3.7 Å away from C4 and C5 of the TCA1 benzothiazole ring, respectively, which make them two potential H‐bond donors if H‐bond acceptors are introduced at these positions (Figure 1 and Figure S1). Indeed, the introduction of nitrogen to the benzothiazole ring (TCA007) improved the IC_50_ by 8‐fold (5±2 nm) compared to TCA1 (48±14 nm) (Figure 1, Table 1, SI methods and Figure S2–S4).

**Table 1:** Correlation between inhibition on DprE1 WT and mutant enzyme activity and cellular potency by TCA1 and analogs (μm).4d

TCA1 analogs	IC_50_ DprE1 WT^[a]^	IC_50_ DprE1 Y321C^[a]^	MIC WT	MIC Y314C
TCA1	0.048±0.014	0.24±0.052	0.23±0.012	5.4±0.32
TCA007	0.0053±0.18	0.13±0.021	0.089±0.0066	2.4±0.34
TCA481	ND^[b]^	ND^[b]^	0.046±0.0025	1.4±0.086
TCA020	0.37±0.13	>10	2.5±0.48	17±1.1
TCA787	0.096±0.038	1.8±0.54	0.44±0.12	14±1.4

[a] All in vitro assays were performed using *M. smegmatis* DprE1 which shows 83 % sequence identity with *Mtb* DprE1. *M. smegmatis* Y321C corresponds to Y314C in *Mtb*. [b] IC_50_ of TCA481 was not determined due to its low solubility under the assay condition.

The thiophene ring is located in a hydrophobic pocket formed by Gly133, Lys367, Phe369 and Asn385 and accepts a H‐bond from His132 Nϵ2 (3.2 Å). Replacement of thiophene with other heterocycles or adding bulky substitutions resulted in reduced activity against *Mtb* (data not shown). Only a fluoro substituent on the thiophene afforded an analog (TCA481) that is 5‐fold more potent than TCA1 based on MIC (Figure 1 and Table 1). To better understand the basis of the activity increase, we solved the crystal structure of DprE1 in complex with TCA481 (Table S1). The structure reveals that the fluoro group forms a H‐bond with Asn385 Nδ2 (3.4 Å). The fluorine is accommodated in the pocket with stronger hydrophobic interactions with residues Phe369, His132, and Lys367 than TCA1 (Figure 2 B and Figure S1).

**Figure 2:**
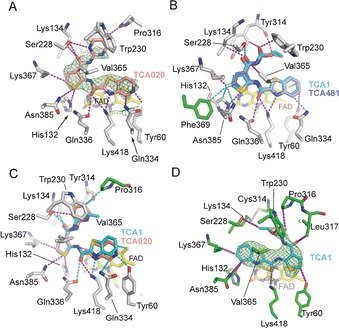
Non‐covalent interactions of TCA inhibitors with DprE1. A) Inhibitor TCA020 (pink) with non‐covalent interactions indicated in dashed lines (magenta). The unbiased Fo−Fc density map (contoured at 2.5 σ) was calculated with model phases prior to incorporation of the ligand in the structural model. Protein residues are shown in gray sticks, FAD in yellow sticks. B, C) Superposition of TCA1 with the inhibitors TCA481 and TCA020, respectively. Dashed lines in cyan indicate new or shortened non‐covalent interactions compared to the TCA1:wt‐DprE1 complex (see Figure S1 for contact distances). Protein side chains in green indicate amino acids that—in TCA1:wt‐DprE1—fall outside the 4 Å contact radius. D) The flipped orientation of TCA1 (cyan) in the DprE1–Y314C active site.

Modification of the carbamate group was carried out; however, most of the analogs such as amides and esters are not active, demonstrating the importance of hydrogen bonding between Ser228 and the carbamate group (3.0 to 3.5 Å) (Figure 2 C and Figure S1). When replacing the carbamate with various heterocycles, only TCA020 with pyrimidine showed moderate activity (MIC=2.5 μm, IC_50_=370±133 nm) (Figure 1 and Table 1). To help optimizing the pyrimidyl substituent, we crystallized DprE1 in complex with TCA020 (SI methods). The DprE1–TCA1 structure revealed that the two nitrogen atoms of the pyrimidine ring occupy the positions of the two oxygens of the carbamate group as H‐bond acceptors. Based on these structural insights, we introduced a methyl group at 4‐position of the pyrimidine to gain a hydrophobic interaction with W230 (TCA787), which has similar activity to TCA1 (Figure 1 and Table 1).

Y314 resides close to the carbamate moiety and it was previously found that a spontaneous TCA1 resistant mutant harbors a Y314C mutation.^6^ We measured the inhibitory activities of TCA1 and its analogs against DprE1 and the Y314C variant and observed 5‐ to 40‐fold shift on IC_50_ values under the same assay condition, which is consistent with the MIC shift between the wildtype (WT) and the mutant strain (Table 1). These data support the notion that growth inhibition of *Mtb* by the TCA1 series of inhibitors is via DprE1 inhibition. We also generated a Y314A mutant and it is sensitive to TCA1 inhibition, suggesting that the side chain of Y314 does not directly contribute to TCA1 binding (Table S2). To elucidate the molecular basis of the resistance mechanism, the structure of DprE1 Y314C bound with TCA1 was determined to 2.2 Å (Figure 2 D, Figure S1 and Table S1). Although the overall fold of the mutant enzyme is identical to wildtype, consequential structural changes in the active site of DprE1 were evident. The most striking difference between the WT and mutant DprE1 complexes is the 180° flipped orientation of the boomerang‐shaped ligand in the active site (Figure S1 and Figure S5). The alternative orientation of the ligand can be linked to the effects of cysteine 314 substitution on the orientation of Lys134, which positions the ϵ‐ammonium group in the void created by substituting the tyrosyl ring with a thiol. The reorientation of Lys134 is driven by formation of a strong H‐bond (2.75 Å) between its ϵ‐ammonium and the thiol of Cys314 and results in a noticeable shape change of the active site around Lys134. Consequently, the acylcarbamate keto‐oxygen in the original orientation would clash with the alkyl moiety of Lys134 (with the shortest distance at 2.7 Å), which likely weakens the binding of TCA1 (Figure S5).

Due to its superior activity against *Mtb* and improved PK properties, TCA007 was selected as a lead compound for further investigation. We evaluated the efficacy of TCA007 against *Mtb* Erdman in acute and chronic BALB/c mouse low dose aerosol infection models.^8^ After treatment for 3 weeks during the acute phase of infection with TCA007 at a dose of 50 mg kg^−1^ and 100 mg kg^−1^ body weight, a dose‐dependent in vivo efficacy was observed with reduction of bacterial load in lung by 1.7 and 2.3 log_10_ CFU, respectively (Figure 3 A and SI methods). This excellent in vivo bactericidal activity is comparable to the first‐line drug rifampin. Following 4 weeks of treatment during the chronic phase of infection, TCA007 at 200 mg kg^−1^ achieved a reduction of >1.7 log_10_ CFU in mouse lungs (Figure 3 B).

**Figure 3:**
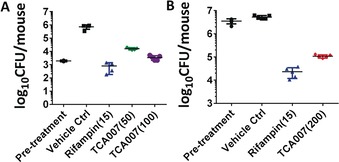
In vivo efficacy study. A) Efficacy of TCA007 in a mouse acute model of established TB. B) Efficacy of TCA007 in a mouse chronic model of established TB. The in vivo dose (mg kg^−1^) of each compound is shown in parentheses.

A typical treatment of TB lasts for more than six months, and therefore, the safety profile of anti‐TB drugs is of great importance. TCA007 is not cytotoxic against Vero and HepG2 cell lines at up to a concentration of 100 μm. TCA007 does not inhibit hERG potassium channel in a patch‐clamp assay (IC_50_>30 μm), suggesting a low risk of cardiotoxicity. In addition, we observed no genetic toxicity in a mini‐Ames mutagenicity test. TCA007 showed no inhibition of four major CYPs, 1A2, 3A4, 2D6, 2C19, but a strong inhibitory effect for CYP2C9 (SI methods).

The potent inhibition of P450 2C9 could reflect oxygenation of thiophene moiety of TCA analogs to an electrophilic S‐oxide or epoxide^9^ that reacts with the enzyme to form an irreversible inhibitor as seen for tienilic acid.^10^ However, the inhibition of 2C9 by TCA analogs was not time dependent indicating a reversible mode (Figure S6). To better understand the interactions of TCA007 with 2C9, the structure of the complex was determined by X‐ray crystallography to 2.0 Å resolution^11^ (PDB: 5W0C) (SI methods, Table S3 and Figure S7). Similar to the binding of TCA1 to DprE1, the thiophene group of TCA007 binds in a hydrophobic pocket of the 2C9 substrate binding cavity that is far from the iron of the heme cofactor where the reactive oxidant is formed, thus preventing oxygenation of the thiophene moiety. The pocket is formed by the inner surface of helix B′ and adjacent residues (Figure 4 A) and hydrophobic residues on helix F across from B′ and helix G above TCA007. Mimicking the role of DprE1 Lys418, Arg108 donates H‐bonds from Nϵ to the carbonyl oxygen of the thiophene carboxamide (3.0 Å) and from Nη2 to the thiazole nitrogen (3.4 Å). The thiophene carbonyl also accepts an H‐bond from a water molecule. The Arg108 Nϵ also contacts the thiophene sulfur. Arg108 plays a critical role in recruiting negatively charged or partially charged substrates such as diclofenac and warfarin, respectively, to the substrate binding site of 2C9. Substitution of Arg108 by alanine,^12^ histidine or phenylalanine^13^ leads to a loss catalytic activity for the two substrates.

**Figure 4:**
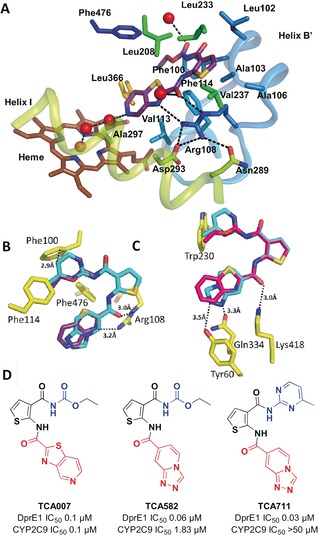
Crystal structure of CYP2C9 with TCA007. A) Key interactions exhibited by TCA007 (violet carbons) in the CYP2C9 active site (PDB:5W0C). Amino acid side‐chains that contact TCA007 are rendered as sticks. The red spheres are water molecules, and the dashed lines depict H‐bonds. B, C) Modeling TCA711 (cyan) in the CYP2C9 structure (overlap with TCA007 in violet) (B) and DprE1 structure (overlap with TCA1 in megenta) (C). D) SAR optimization strategy for TCA1.

Phe100, Leu102, and Phe114 form a hydrophobic pocket together with Phe476 where the carbamate group of TCA007 is bound (Figure 4 A). In contrast to DprE1, protein H‐bond donors for the carbamate are not present in this pocket, but the cavity is open to solvent and an ordered water molecule donates an H‐bond to the carbonyl of the acyl group. The pyridyl nitrogen of the pyridothiozole group is oriented toward the heme but is too distant (6.6 Å) for coordination to the heme iron. Consistent with this observation, TCA007 does not significantly alter the visible absorption spectrum of 2C9 in solution. In contrast, miconazole binds to the heme iron of 2C9 and alters the spectrum of the heme co‐factor, and competitive displacement of iron bound miconazole by TCA007 reverts the spectrum of the miconazole complex to that of the apo enzyme (Figure S8). This portion of the active site is also hydrophobic, but a molecule of water is evident 2.88 Å from the pyridyl nitrogen of TCA007 (Figure 4 A). The water molecule is hydrogen bonded to a second water molecule that donates a lone pair of electrons to the heme iron and a H‐bond to the backbone carbonyl of Ala297. In general, the extensive hydrophobic interactions and hydrogen bonding interactions with Arg108 are likely to form the basis for the high affinity, reversible binding of TCA007 to 2C9, but these interactions also sequester TCA007 in position that reduces the likelihood of metabolism, which is consistent with absent or low rates of metabolite formation or time dependent inhibition.

Removal of these aforementioned heteroatoms will likely reduce the CYP2C9 inhibition. However, it was shown that the thiophene group and carbonyl group are also essential for DprE1 binding. Therefore, we instead modified the ring A to reduce CYP2C9 inhibition while gaining extra interactions in ring B to maintain DprE1 binding (Figure 4 B). Both sulfur and nitrogen in the ring A were removed to reduce the interaction in CYP2C9, and two nitrogens were introduced into ring B which potentially form H‐bonds with Tyr60 and Glu334 to compensate for the reduced interaction in DprE1 (Figure 4 C). The IC_50_ ratio (CYP2C9 over DprE1) for TCA582 and TCA711 were 33 and 1515, respectively, which, compared with TCA007 (ratio=1.8), represents a significant improvement in selectivity (Figure 4 D). The modeling of CYP2C9 with both TCA582 and TCA711 revealed that the carbamate group could gain favorable hydrophobic interactions with Phe100 (3.6 Å), Phe114 (4.1 Å) and Phe476 (3.8 Å). These interactions would be weakened by an unfavorable hydrophobic interaction with Phe100 (2.9 Å and 3.0 Å) when replacing the carbamate with pyrimidyl substituent, which could explain the nearly eliminated inhibition of CYP2C9 by TCA711 (IC_50_ of CYP2C9 >50 um) (Figure 4 D). The in vivo studies on the advanced analog TCA711 are ongoing.

In summary, we demonstrate the mode of inhibition of DprE1 by the thiophene scaffold via enzymatic and structural studies. The SAR studies not only helped us to optimize the potency of the inhibitors but revealed an unconventional resistant mechanism of DprE1. The optimized lead compound TCA007 showed excellent in vitro and in vivo activities, PK properties, and low risk of toxicity profile except for the strong inhibition of CYP2C9. Guided by the structures of CYP2C9 in complex with TCA1 analogs, an optimized molecule, TCA711, was generated with differential inhibitory activities against DprE1 and CYP2C9, which paved the road for further development of a clinical candidate to treat TB.

## Conflict of interest

The authors declare no conflict of interest.

## Supporting information

As a service to our authors and readers, this journal provides supporting information supplied by the authors. Such materials are peer reviewed and may be re‐organized for online delivery, but are not copy‐edited or typeset. Technical support issues arising from supporting information (other than missing files) should be addressed to the authors.

Supplementary
