# Supplementary material for: Determinants of the Inhibition of DprE1 and CYP2C9 by Antitubercular Thiophenes
Source: Angew Chem Int Ed Engl. 2017 Sep 7;56(42):13011–5. doi: 10.1002/anie.201707324 (PMC5659129; doi:10.1002/anie.201707324)
Supplement: Supplementary file 1 — Supplementary [file ANIE-56-13011-s001.pdf]

Angewandte Chemie
Eine Zeitschrift der Gesellschaft Deutscher Chemiker

Supporting Information

Determinants of the Inhibition of DprE1 and CYP2C9 by Antitubercular Thiophenes

*Renhe Liu[+], Xiaoxuan Lyu[+], Sarah M Batt, Mei-Hui Hsu, Michael B Harbut, Catherine Vilchèze, Bo Cheng, Kehinde Ajayi, Baiyuan Yang, Yun Yang, Hui Guo, Changyou Lin, Fei Gan, Chen Wang, Scott G. Franzblau, William R. Jacobs, Jr., Gurdyal S. Besra, Eric F. Johnson, Mike Petrassi, Arnab K. Chatterjee, Klaus Fütterer, and Feng Wang**

anie_201707324_sm_miscellaneous_information.pdf

Table of Contents

Materials and Methods……………………………………………………………..…... S2
General methods……………………..…………………………………………..…… S2
Synthesis and characterization data for TCA analogs…………...……………….……… S3
MICs measurement……………………………………………………………....….. S6
Expression and purification of M. smegmatis DprE1 and the mutants….……….….…… S6
DprE1ctivity and inhibition studies……………………..………………….…….….… S7
Expression of M. tuberculosis DprE1 for Crystallization ………………….….….……… S7
In vivo efficacy in mice……….……………………………….…..…………………… S8
Pharmacokinetics …………….……..…………………………….……..…...…… S8
Inhibition of CYPs……………………………………………………...……………..… S8
In vitro cytotoxicity assay ……………………………………………..…………….… S8
Expression of Cytochrome P450 2C9 for Crystallization…………………….….…..….. ..S9
References…………………………..………………………………………...….. S11

Supplementary Figures…………………………………………………….…….……… S12
Figure S1. ……………………………………………………………….………..... S12
Figure S2. ……………………………………………………………….………..… S13
Figure S3. ……………………………………………………………….…………… S13
Figure S4. ……………………………………………………………….………..… S13
Figure S5. ……………………………………………………………….…….……… S14
Figure S6. ……………………………………………………………….…….……… S15
Figure S7. ……………………………………………………………….…….……… S15
Figure S8. .... S16

Supplementary Tables…………………………..……………………………….……… S17
Table S1. ……………………………………………………………….………………S17
Table S2. ……………………………………………………………………….……..… S18
Table S3. …………………………………………………………………..………..… S18

^{1}H and ^{13}CNMR…………………………………………………………….…..…… S19

References in main text……………………….……………………………...….. S27

Materials and Methods:

General Methods

All reactions in non-aqueous media were conducted under a positive pressure of dry nitrogen in glassware that had been oven dried prior to use unless noted otherwise. Anhydrous solutions of reaction mixtures were transferred via an oven dried syringe or cannula. All solvents and reagents that were purchased commercially (either Sigma Aldrich or Thermo Fisher) were used without further purification unless otherwise specified. Reagents were purchased from Aldrich, Acros, Combi block, or Enamine unless otherwise noted. Analytical thin-layer chromatography was conducted on EMD Chemicals Silica gel 60 F254 plates, and compounds were visualized with UVG-11 mineralight UV lamp (UVP, Inc) at 254 nm. Flash column chromatography was performed with silica gel (Combi). ^{1}H and ^{13}C Nuclear Magnetic Resonance (NMR) spectra were obtained on a Bruker-400 UltraShield recorded in ppm (δ) downfield of TMS (δ = 0) in $CDCl_3$, CD_3OD, d-DMSO. Signal splitting patterns were described as singlet (s), doublet (d), triplet (t), quartet (q), quintet (quint), or multiplet (m), with coupling constants (*J*) in hertz. High resolution mass spectra (HRMS) were performed on an Electron Spray Injection (ESI) mass spectrometer. Liquid chromatographymass spectrometry (LC-MS) was performed on Agilent 1100 series equipped with eclipse plus C18 analytical column (5µm, 4.6×50 mm). The elution condition was a linear gradient increase of solvent B from 10% to 90% over a time period of 6 minutes at a flow rate of 1 mL/min.

Synthesis and characterization data for TCA analogs:
Unless noted otherwise, the synthesis of TCA1 and other analogs were reported. [9]

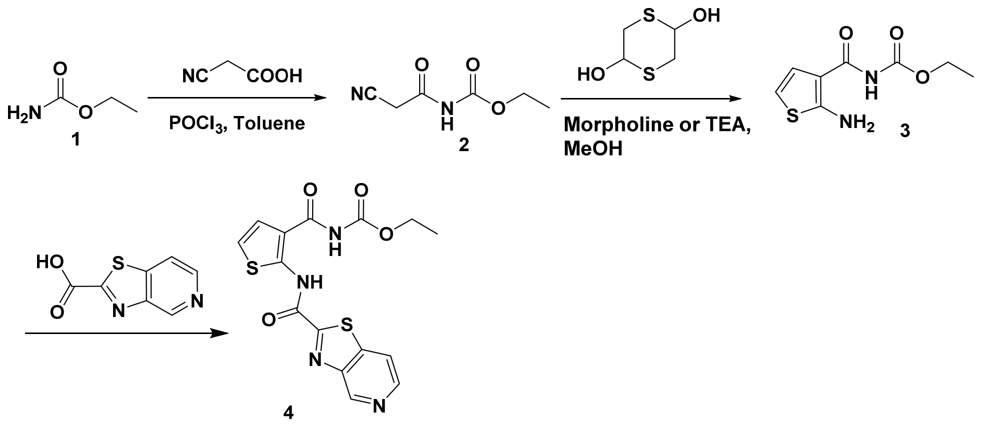

Ethyl carbamate (1.14 g, 12.8 mmol) and cyanoacetic acid (1.10 g, 12.9 mmol) were suspended in toluene (4 mL). The mixture was treated with phosphorus oxachloride (0.63 mL, 6.74 mmol) and DMF (0.31 mL) and heated at 75 oC for 1.5 hrs. The reaction was cooled to room temperature and poured into an ice-water (20 mL) and stirred for 30 min. The precipitation was collected and washed with H2O and was thoroughly dried to give the target carbamate 2 (1.2 g, 55 %). The desired carbamate was directly used for the next step without further purification.

Ethyl (2-cyanoacetyl) carbamate (1.56 g, 10.0 mmol), 1, 4-dithiane-2, 5-diol (800 mg, 5.0 mmol) and morpholine (0.87 mL, 10.0 mmol) were mixed in MeOH (10 mL) at room temperature under argon. The mixture was heated at 65 oC for 3 hrs and poured into water (200 mL) and extracted with Et2O (150 mL x 4) followed by washing with sat. NH4Cl solution (150 mL x 3). The organic phase was dried with sodium sulfate and concentrated in vacuo. The residue was recrystallized with EtOAC / Hexanes to give desired thiophene 3 (1.0 g, 47 %). In some cases, product was purified by silica column chromatography with MeOH/DCM.

Benzothiazole-2-carboxylic acid (53 mg, 0.3 mmol) was suspended in dichloromethane (1.5 mL) and cooled using ice-water bath. Oxalyl chloride (28.0 µL, 0.33 mmol) was added followed by a drop of DMF. After gas evolution stopped, the reaction became a clear yellowish solution and further cooled to -30 oC. To this solution, pyridine (78 µL, 0.75 mmol) was added followed by the solution of thiophene amine (65 mg, 0.3 mmol) in dichloromethane (1.0 mL). The reaction mixture was allowed to warm up to room temperature for 30 min and then quenched by methanol (0.5 mL). The reaction mixture was poured into water (5 mL) and extracted with a mixed solvent ($CHCl_3$: i-PrOH 4:1, 5mL x 2). The organic phase was washed with sat. NH4Cl solution (5 mL) and dried with sodium sulfate. The solution was concentrated under vacuum to give a solid which was purified by silica column chromatography (MeOH/DCM) to yield the title compound. ^{1}HNMR (400MHZ, DMSO-d6) 13.13 (s, 1H), 10.90 (s, 1H), 9.60 (s, 1H), 8.70 (d, J = 8.0 Hz, 1H), 8.38 (d, J = 4 Hz, 1H), 7.79 (d, J = 4 Hz, 1H), 7.23 (d, J = 4 Hz, 1H), 4.302 (q, J = 8 Hz, 2H), 1.370 (t, J = 8 Hz, 3H). ^{13}C NMR (101 MHz, DMSO-d6) δ 163.95, 163.90,

156.63, 151.58, 149.51, 147.51, 147.09, 145.47, 144.67, 124.01, 118.59, 118.29, 116.99, 61.81, 14.69. HRMS (ESI-TOF) [M+H] calculated for $[C_{15}H_{13}N_4O_4S_2]^+$ 377.0378, observed 377.0392.

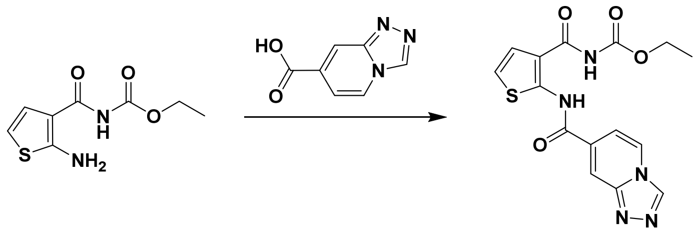

Benzothiazole-2-carboxylic acid (49 mg, 0.3 mmol) was suspended in dichloromethane (1.5 mL) and cooled using ice-water bath. Oxalyl chloride (28.0 µL, 0.33 mmol) was added followed by a drop of DMF. After gas evolution stopped, the reaction became a clear yellowish solution and further cooled to -30 °C. To this solution, pyridine (78 µL, 0.75 mmol) was added followed by the solution of thiophene amine (65 mg, 0.3 mmol) in dichloromethane (1.0 mL). The reaction mixture was allowed to warm up to room temperature for 30 min and then quenched by methanol (0.5 mL). The reaction mixture was poured into water (5 mL) and extracted with a mixed solvent (CHCl3: i-PrOH 4:1, 5mL x 2). The organic phase was washed with sat. NH_4Cl solution (5 mL) and dried with sodium sulfate. The solution was concentrated under vacuum to give a solid which was purified by silica column chromatography (MeOH/DCM) to yield the title compound. ^{1}HNMR (400MHZ, DMSO-d6) ^{1}H NMR (400 MHz, DMSO-*d*$_6$) δ 12.66 (s, 1H), 10.88 (s, 1H), 9.46 (s, 1H), 8.77 (d, *J* = 7.2 Hz, 1H), 8.37 (s, 1H), 7.74 (d, *J* = 5.9 Hz, 1H), 7.37 (dd, *J* = 7.2, 1.6 Hz, 1H), 7.15 (d, *J* = 5.9 Hz, 1H), 4.23 (q, *J* = 7.1 Hz, 2H), 1.30 (t, *J* = 7.1 Hz, 3H). ^{13}C NMR (101 MHz, DMSO-d6) δ 164.32, 161.68, 158.62, 151.65, 139.37, 137.95, 126.63, 123.95, 123.89, 117.68, 116.37, 115.73, 111.49, 61.82, 14.70. HRMS (ESI-TOF) [M+H] calculated for $[C_{15}H_{14}N_5O_4S]^+$ 360.0766, observed 360.0751.

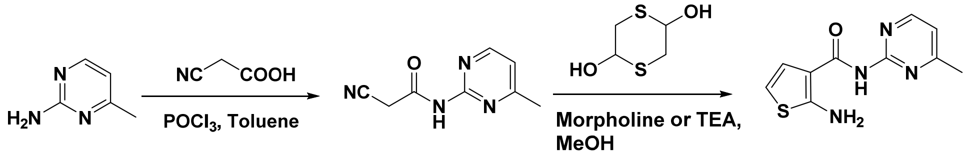

Ethyl carbamate (1.14 g, 12.8 mmol) and cyanoacetic acid (1.10 g, 12.9 mmol) were suspended in toluene (4 mL). The mixture was treated with phosphorus oxachloride (0.63 mL, 6.74 mmol) and DMF (0.31 mL) and heated at 75 oC for 1.5 hrs. The reaction was cooled to room temperature and poured into an ice-water (20 mL) and stirred for 30 min. The precipitation was collected and washed with H2O and was thoroughly dried to give the target carbamate 2 (1.2 g, 55 %). The desired carbamate was directly used for the next step without further purification.

Ethyl (2-cyanoacetyl) carbamate (1.56 g, 10.0 mmol), 1, 4-dithiane-2, 5-diol (800 mg, 5.0 mmol) and morpholine (0.87 mL, 10.0 mmol) were mixed in MeOH (10 mL) at room temperature under argon. The mixture was heated at 65 oC for 3 hrs and poured into

water (200 mL) and extracted with Et2O (150 mL x 4) followed by washing with sat. NH4Cl solution (150 mL x 3). The organic phase was dried with sodium sulfate and concentrated in vacuo. The residue was recrystallized with EtOAC / Hexanes to give desired thiophene 3 (1.0 g, 47 %). In some cases, product was purified by silica column chromatography with MeOH/DCM.

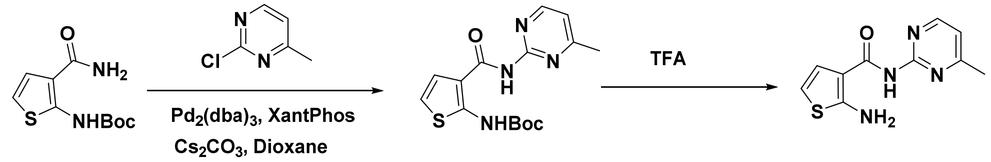

A mixture of 2-chloro-6-methylpyrimidine (1.39 g, 10.8 mmol), 2-Boc-amino-thiophene-3-acetamide (2.4 g, 10 mmol), tris(dibenzylideneacetone)dipalladium(0) (0.20 g, 0.22 mmol), 4,5-bis(diphenylphosphino)-9,9-dimethylxanthene (0.37 g, 0.65 mmol), cesium carbonate (4.2 g, 12.9 mmol) and 1,4-dioxane (30 mL) is heated by microwave irradiation at 150°C for 2.5 hr. After cooling to room temperature, the mixture is filtered through a pad of celite. The filtrate is concentrated under reduced pressure and the residue is purified by silica gel chromatography (3:1 hexanes/ethyl acetate) to achieve the titled product.

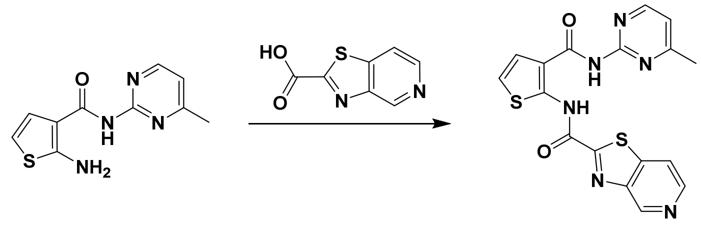

Benzothiazole-2-carboxylic acid (53 mg, 0.3 mmol) was suspended in dichloromethane (1.5 mL) and cooled using ice-water bath. Oxalyl chloride (28.0 µL, 0.33 mmol) was added followed by a drop of DMF. After gas evolution stopped, the reaction became a clear yellowish solution and further cooled to -30 ºC. To this solution, pyridine (78 µL, 0.75 mmol) was added followed by the solution of thiophene amine (70 mg, 0.3 mmol) in dichloromethane (1.0 mL). The reaction mixture was allowed to warm up to room temperature for 30 min and then quenched by methanol (0.5 mL). The reaction mixture was poured into water (5 mL) and extracted with a mixed solvent (CHCl3: i-PrOH 4:1, 5mL x 2). The organic phase was washed with sat. NH4Cl solution (5 mL) and dried with sodium sulfate. The solution was concentrated under vacuum to give a solid which was purified by silica column chromatography (MeOH/DCM) to yield the title compound as yellow powder. ^{1}H NMR (400 MHz, DMSO-d6) 13.36 (s, 1H), 10.96 (s, 1H), 9.56 (s, 1H), 8.68 (d, J = 8.0 Hz, 1H), 8.66 (d, J = 4.0 Hz, 1H), 8.37 (d, J = 4.0 Hz, 1H), 7.90 (d, J = 8.0 Hz, 1H), 7.75 (q, J = 4.0 Hz, 2H), 2.50 (s, 3H). ^{13}C NMR (101 MHz, DMSO-d6) δ168.91, 164.27, 163.89, 158.42, 157.70, 156.58, 147.05, 146.39, 145.44, 144.70, 124.22,

118.38, 117.82, 117.77, 24.00. HRMS (ESI-TOF) [M+H] calculated for $[C_{17}H_{13}N_6O_2S]^+$ 397.0541, observed 397.0556.

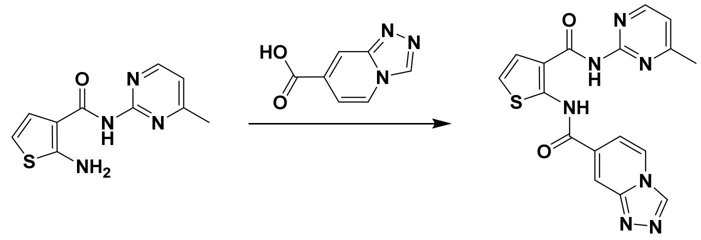

Benzothiazole-2-carboxylic acid (53 mg, 0.3 mmol) was suspended in dichloromethane (1.5 mL) and cooled using ice-water bath. Oxalyl chloride (28.0 µL, 0.33 mmol) was added followed by a drop of DMF. After gas evolution stopped, the reaction became a clear yellowish solution and further cooled to -30 oC. To this solution, pyridine (78 µL, 0.75 mmol) was added followed by the solution of thiophene amine (65 mg, 0.3 mmol) in dichloromethane (1.0 mL). The reaction mixture was allowed to warm up to room temperature for 30 min and then quenched by methanol (0.5 mL). The reaction mixture was poured into water (5 mL) and extracted with a mixed solvent (CHCl3: i-PrOH 4:1, 5mL x 2). The organic phase was washed with sat. NH4Cl solution (5 mL) and dried with sodium sulfate. The solution was concentrated under vacuum to give a solid which was purified by silica column chromatography (MeOH/DCM) to yield the title compound. ^{1}HNMR (400MHZ, DMSO-d6) 13.05 (s, 1H), 10.93 (s, 1H), 9.45 (s, 1H), 8.75 (d, J = 8 Hz, 1H), 8.63 (d, J = 8 Hz, 1H), 8.33 (s, 1H), 7.86 (d, J = 4 Hz, 1H), 7.36 (d, J = 4 Hz, 1H), 7.20 (d, J = 4 Hz, 1H), 7.16 (d, J = 4 Hz, 1H), 2.50 (m, 1H). ^{13}C NMR (101 MHz, DMSO-d6) δ 168.85, 164.39, 161.33, 158.39, 157.68, 148.39, 147.86, 137.90, 131.89, 126.60, 123.96, 117.69, 117.56, 116.77, 115.54, 111.40, 23.98. HRMS (ESI-TOF) [M+H] calculated for $[C_{17}H_{14}N_7O_2S]^+$ 380.0930, observed 380.0932.

MICs measurement. MICs were determined using the microplate alamar blue assay (MABA). Cultures were incubated in 200 uL 7H12 medium together with the test compound in 96-well plates for 7 days at 37°C. Alamar Blue and Tween 80 were added, and incubation was continued for 24 h at 37°C. Fluorescence was determined at excitation/emission wavelengths of 530/590 nm, respectively. The MIC was defined as the lowest concentration effecting a reduction in fluorescence of 90% relative to that in the controls. (Figure S3)

Expression and purification of M. smegmatis DprE1 and the mutants. The M. smegmatis DprE1 WT and mutants were expressed and purified as previously reported.[1] Protein was expressed with an N-terminal His6-thioredoxin tag in E coli BL21(DE3)-pLysS. The E. coli strain was cultured in LB medium at 37°C for 3 hours and then induced with 1mM IPTG at 16°C for overnight. Cells were harvested by centrifugation and resuspended in 50 mM Tris-HCl pH 8.0, 500 mM NaCl, 1 mM MgCl2, 10% (v/v) glycerol, 1% Triton X-100 buffer containing complete EDTA-free protease inhibitors cocktail, Roche). Cell disruption was carried out by sonication and the clear lysate was obtained by centrifugation at 16000 rpm for 1 h at 4°C. The supernatant was loaded onto a Ni-NTA column (Qiagen). The column was washed twice with 5 ml washing buffer (50

mM Hepes, pH 8.0, 500 mM NaCl, 1 mM MgCl2 and 20 mM imidazole) and then eluted with elution buffer (50 mM Tris, pH 8.0, 500 mM NaCl, 1 mM MgCl2 and 250 mM imidazole). The purified His-tagged samples were then buffer exchanged to 50 mM Tris-HCl, 250 mM NaCl, 1 mM MgCl2 and removed His-tag by Pierce™ HRV 3C Protease (Thermo Scientific). The NaCl concentration was adjusted to 500 mM and the cleaved tag and protease were removed by running the protein solution through a 5 mL Ni-NTA column. The protein solution was concentrated and loaded to size exclusion chromatography using an AKTA Explorer FPLC system and a Superdex 75 HR column. The fractions were collected, concentrated and flash-frozen in liquid nitrogen for storage at -80°C. The concentration of DprE1 were determined by absorbance at 280 nm and the yield is about 5mg/L.

DprE1 activity and inhibition studies. DprE1 assays were performed as described previously.[2] Briefly, a 30 µL reaction was performed at 30 °C in 384-well black plates (catalog no. 3573, Corning Costar, Corning, NY) in buffer containing 50mM Hepes, pH 7.5, 100 mM NaCl, 1.5% (v/v) DMSO, 100 µM Tween-20, 2 µM FAD, and 50 µM resazurin, with variable concentrations of FPR and DprE1 either wild type or Y321C or Y321A mutant. Reactions were monitored by following an increase in fluorescence intensity (λex = 530 nm, λem = 595 nm) associated with the formation of resorufin. Steady-state kinetic studies were carried out using a microplate reader Spectramax M5 (Molecular Devices Corporation, California, USA). For inhibition studies, DprE1 WT or mutant (1 uM) was measured with the resazurin assay with 1 mM FPR in the presence of different inhibitor concentrations (0 to 50 uM; all inhibitors were dissolved in dimethyl sulfoxide to afford solutions with different concentrations and 1% dimethyl sulfoxide final concentration fixed under all assay conditions). The IC50 values were obtained by plotting the initial velocities with inhibitor concentration.

M. tuberculosis DprE1 expression and purification. DprE1 (Rv3790) was coexpressed with chaperones from E. coli (GroES) and M. tuberculosis (CPN60.2) in E. coli BL21 (DE3). Liquid cultures of E. coli cells transformed with pCDFDuet-Rv3790 and pTrc 60.2-GroES, were grown at 37 °C in LB broth (Difco) supplemented with kanamycin (50 µg/mL) and ampicillin (100 µg/mL). The culture temperature was reduced to 16ºC at OD_{600} of 0.4–0.6, and protein expresssion induced by adding 0.5 mM isopropylthiogalactoside, followed by incubation under shaking (16ºC, 14 – 16 h). Cells were harvested, washed with 0.85% saline and frozen (−20 °C). Frozen cell pellets were thawed and resuspended in 30 ml buffer A (50 mM NaH_2PO_4, 300 mM NaCl, 10 mM imidazole, pH 8), supplemented with EDTA-free protease inhibitor (Roche). Cells were lysed by sonication (Sonicator Ultrasonic Liquid Processor XL; Misonix, 20-s pulses, 40-s cooling, 10 min total, 4 ºC) and centrifuged (27,000 × g, 40min, 4 °C), and the supernatant passed through a preequilibrated (buffer A) Ni-NTA column (1 ml bed volume, His-Trap, GE Healthcare). The column was washed with 50 ml of buffer A plus 20 mM imidazole, and the protein was eluted with a 50–300 mM gradient of imidazole. Fractions containing protein were dialyzed into buffere B (20 mM Tris·HCl pH 8.5, 10 mM NaCl, 10% (v/v) glycerol), and loaded onto a QHP ion exchange column (1 ml bed volume, Amersham), washed with 10 mL of buffer B plus 50 mM NaCl, and eluted with

a NaCl step gradient (20 mM increments) from 100 mM to 200 mM. Fractions containing pure protein were dialyzed overnight into buffer B and concentrated by ultrafiltration (35 mg/ml, Amicon Ultra, MWCO 10,000).

M. tuberculosis DprE1 Crystallization and Structure Determination. Crystals of DprE1 were grown by sitting drop vapor diffusion in 96-well plates (Swissci), pipetting crystallization drops (150 nl protein + 150 nl reservoir) using a Mosquito liquid handling robot (TTP Labtech). Crystals grew at previously established conditions [3], with the reservoir containing 0.2 M imidazole (pH 7.5) and 30 – 35 % (v/v) polypropylene glycol 400. Crystals appeared after 1–3 d and could be mounted from the drop and frozen in liquid nitrogen without additional cryoprotection. Diffraction data were recorded on Diamond Light Source, beamlines I02 and I03, and reduced using XDS, XSCALE[4] (**Table S1**), and phased by molecular replacement, using the structure of apo-DprE1 (PDB entry 4FEH, Batt et al. 2012) as a search model. Building and refinement used software packages COOT[5], REFMAC5[6], and PHENIX.REFINE[7]. Figures were prepared using PyMol (www.pymol.org), adopting the Corey-Pauling-Koltun (CPK) coloring scheme: O, red; N, blue; S, sulfur; and C, as indicated in figure legends.

In vivo efficacy in mice. Female 20g BALB/c mice were infected by aerosol with a low dose of M. tuberculosis Erdman. The protocol results in the deposition of approximately 50 to 100 bacilli into the lungs, and the course of infection was then followed by plating homogenates of the lungs on 7H11 agar and determining CFU. Controls consisted of mice treated with the vehicle only. The compounds were prepared weekly by suspension in 0.5% (wt/vol) carboxymethylcellulose (CMC) such that the target dosages were obtained by once-daily dosing by oral gavage of a 200 mL suspension. Groups of 5 mice were dosed for 5 consecutive days each week. The suspensions were stored at 4°C between daily doses. Mice were sacrificed 3 days after the final dose to minimize carryover from the lung homogenates to the plating medium. Both lungs were homogenized and diluted in Hanks' balanced salt solution (HBSS)-Tween, and aliquots were plated on Middlebrook 7H11 medium. CFU were determined after 3 weeks of incubation at 37°C. For statistical analysis of efficacy data, multiple comparisons among pairs were performed by the Bonferroni method.

Pharmacokinetics. Female BALB/c mice were administered TCA007, formulated in 0.5% MC and 0.5% Tween, at 50 and 100 mg/kg by oral gavage. Plasma samples were collected from three mice per time point over the course of 24 h using sodium heparin as an anticoagulant. In a separate study, mice were administered 5 mg/kg TCA007 (formulated in 25% PEG300) by intravenous (i.v.) bolus, and blood samples were collected from three mice per time point over the course of 24 h into potassium EDTA-containing tubes. Plasma concentrations were determined by LC-MS/MS.

Inhibition of CYPs. TCA analogs were incubated with individual CYP isoforms at 37°C for 20 to 30 min in pooled HLM (0.25 mg protein/ml), phosphate buffer (100 mM, pH 7.4), magnesium chloride (MgCl2) (5 mM), NADPH (1 mM), and CYP probe substrates (at the approximate Km). The CYP probe substrates and reactions were phenacetin dealkylation for CYP1A2, coumarin 7-hydroxylation for CYP2A6, bupropion

hydroxylation for CYP2B6, paclitaxel 6'-hydroxylation for CYP2C8, diclofenac 4'-hydroxylation for CYP2C9, S-mephenytoin 4'-hydroxylation for CYP2C19, bufuralol 1'-hydroxylation for CYP2D6, chlorzoxazone 6-hydroxylation for CYP2E1, and midazolam 1'-hydroxylation and testosterone 6-hydroxylation for CYP3A4. Experiments were performed in duplicate. Following the addition of acetonitrile and centrifugation at 3,000 to 4,000 rpm for 10 min at 4°C to remove protein, supernatants were analyzed for the formation of CYP probe metabolites by LC-MS/MS, and the percent inhibition was estimated. To determine the 50% inhibitory concentrations (IC50s) of TCA analogs inhibition of CYP2C8, CYP2C19, and CYP3A4 (with two substrates, midazolam and testosterone), TBA-354 (0.01, 0.1, 1, 10, and 30 M) was incubated with HLM (0.3 mg/ml) and NADPH (1 mM). After 30 min of incubation at 37°C, formation of the metabolite of the CYP-specific substrate in the absence and presence of test compound was measured. Ketoconazole was used as a positive control for CYP3A4 inhibition. For assessment of time-dependent inhibition, HLM were preincubated with TBA-354 for 30 min in the presence of NADPH before the addition of probe substrates to measure CYP activity (preincubation mixture not diluted).

In vitro cytotoxicity assay. Vero epithelial cells (from African green monkey; ATCC CCL-81) were cultured in Dulbecco's modified Eagle's medium (DMEM) supplemented with 10% fetal bovine serum (FBS) and maintained in a humidified incubator (37°C in 5% CO_2). Cells were dislodged with a cell scraper, collected by centrifugation, resuspended in fresh medium at 10^6 cells/ml, dispensed into 96-well microtiter plates (100 ml/well), and incubated for 18 h at 37°C. Two-fold serial dilutions of test compounds in DMEM with FBS were subsequently added, and cells were incubated for another 72 h. In triplicate studies, the cytopathic effects of compounds were evaluated colorimetrically by using the 3-(4, 5- dimethyl-2-thiazolyl)-2, 5-diphenyl-2H-tetrazolium bromide (MTT) cell proliferation assay (ATCC).

Expression of Cytochrome P450 2C9 for Crystallization. A modified protein, P450 2C9dH, was used for crystallization as described previously for determination of the structure of the 2C9 flurbiprofen complex.[8] The protein was modified by replacing codons for native residues 2-22 encoding transmembrane helix with those encoding the sequence AKKT. The C-terminus was modified also by replacing the native C-terminal valine residue with the sequence IHHH to facilitate purification of the protein. The P450 2C9dH expression plasmid was co-transformed with pGro7 (Takara) in Escherichia coli strain DH5a, and the expressed protein was isolated by column chromatography employing nickel-nitrotriloacetate agarose and carboxymethyl Sepharose Fast Flow as described previously.

Crystallization of P450 2C9dH. The protein was crystallized by hanging drop vapor diffusion. The purified protein (0.6 mM) in 50 mM KPi, pH 7.4, 0.5M NaCl, 20% glycerol, 1.0 mM EDTA, and 0.2 mM dithiothreitol was combined 9:1 (v/v) with 50mM flurbiprofen in MeOH. This mixture was combined 1:0.25:1.25 (v/v/v) with aqueous 24 mM Cymal-5 and the precipitin/well solution that contained 0.1 M HEPES, pH 8.5, 2% PEG 3350. The 2C9 TCA007 complex was generated by transferring crystals of the 2C9 flurbiprofen complex into an exchange buffer containing TCA007. The exchange buffer

was prepared by mixing the protein buffer (without protein, flurbiprofen, or DTT), reservoir solution (containing 0.1M HEPES, pH 8.5, 16% PEG3350), 24mM Cymal-5, 30 mM TCA007 in DMSO, and MeOH with the following proportions by volume, 18:25:5:1:1. Crystals were transferred 10 times to fresh exchange buffer over a 5 day period to remove the flurbiprofen and saturate 2C9dH with TCA007. The crystals were soaked briefly in a cryo buffer containing 0.1 M HEPES, pH 8.5, 12.5% PEG3350, 30% glycerol, 50 mM NaCl and 1mM TCA007 in DMSO (the final DMSO is 2%) before rapid freezing in liquid nitrogen prior shipping to the Stanford Synchrotron Radiation Lightsource Beam Line 9-2 for remote data collection.

P450 2C9dH Structure determination. The structure of the 2C9 TCA007 complex was determined using data collected from a single crystal at 100K using. The data were integrated using XDS[4] and scaled using Aimless[10] to a limiting resolution of 2.0 Å in space group H3. Data processing statistics are reported in Table S3. Initial phases were determined by molecular replacement using the protein chain of the 1R9O structure of 2C9dH flurbiprofen complex which identified a single protein chain in the unit cell. Coot[5] was used to adjust and build portions of the model. Phenix[7] was used for weighted reciprocal space refinement of the atomic coordinates and atomic B-values against the reflections with geometry restraints. Refinement statistics and quality assessments are reported for the final model in Table S3. The model is complete from residue 27 to 490 of the native protein. The orientation and position of the TCA007 in the active site was well determined by a 2DFo-mFc electron density map as illustrated by a ligand omit map in Figure S7.

References

[1] S. M. Batt, M. Cacho Izquierdo, J. Castro Pichel, C. J. Stubbs, L. Vela-Glez Del Peral, E. Perez-Herran, N. Dhar, B. Mouzon, M. Rees, J. P. Hutchinson, R. J. Young, J. D. McKinney, D. Barros Aguirre, L. Ballell, G. S. Besra, A. Argyrou, *ACS Infect Dis* **2015**, *1*, 615-626.

[2] J. Neres, F. Pojer, E. Molteni, L. R. Chiarelli, N. Dhar, S. Boy-Rottger, S. Buroni, E. Fullam, G. Degiacomi, A. P. Lucarelli, R. J. Read, G. Zanoni, D. E. Edmondson, E. De Rossi, M. R. Pasca, J. D. McKinney, P. J. Dyson, G. Riccardi, A. Mattevi, S. T. Cole, C. Binda, *Sci Transl Med* **2012**, *4*, 150ra121.

[3] S. M. Batt, T. Jabeen, V. Bhowruth, L. Quill, P. A. Lund, L. Eggeling, L. J. Alderwick, K. Futterer, G. S. Besra, *Proceedings of the National Academy of Sciences of the United States of America* **2012**, *109*, 11354-11359.

[4] W. Kabsch, *Acta Crystallogr D Biol Crystallogr* **2010**, *66*, 125-132.

[5] P. Emsley, B. Lohkamp, W. G. Scott, K. Cowtan, *Acta Crystallogr D Biol Crystallogr* **2010**, *66*, 486-501.

[6] G. N. Murshudov, A. A. Vagin, E. J. Dodson, *Acta Crystallogr D Biol Crystallogr* **1997**, *53*, 240-255.

[7] P. V. Afonine, R. W. Grosse-Kunstleve, N. Echols, J. J. Headd, N. W. Moriarty, M. Mustyakimov, T. C. Terwilliger, A. Urzhumtsev, P. H. Zwart, P. D. Adams, *Acta Crystallogr D Biol Crystallogr* **2012**, *68*, 352-367.

[8] M. R. Wester, J. K. Yano, G. A. Schoch, C. Yang, K. J. Griffin, C. D. Stout, E. F. Johnson, *J Biol Chem* **2004**, *279*, 35630-35637.

[9] A. K. Chatterjee, F.Wang, P. G. Schultz, K. Ajayi, J. Wang, R. Halder, P. Kumar, B. Yang, R. Liu, B. Cheng, T. Kaneko, **2014**, WO/2014/190199

[10] Evans, Philip R., and Garib N. Murshudov. *Acta Crystallographica Section D Biological Crystallography* **2013,** 69, 1204-1214.

Figures

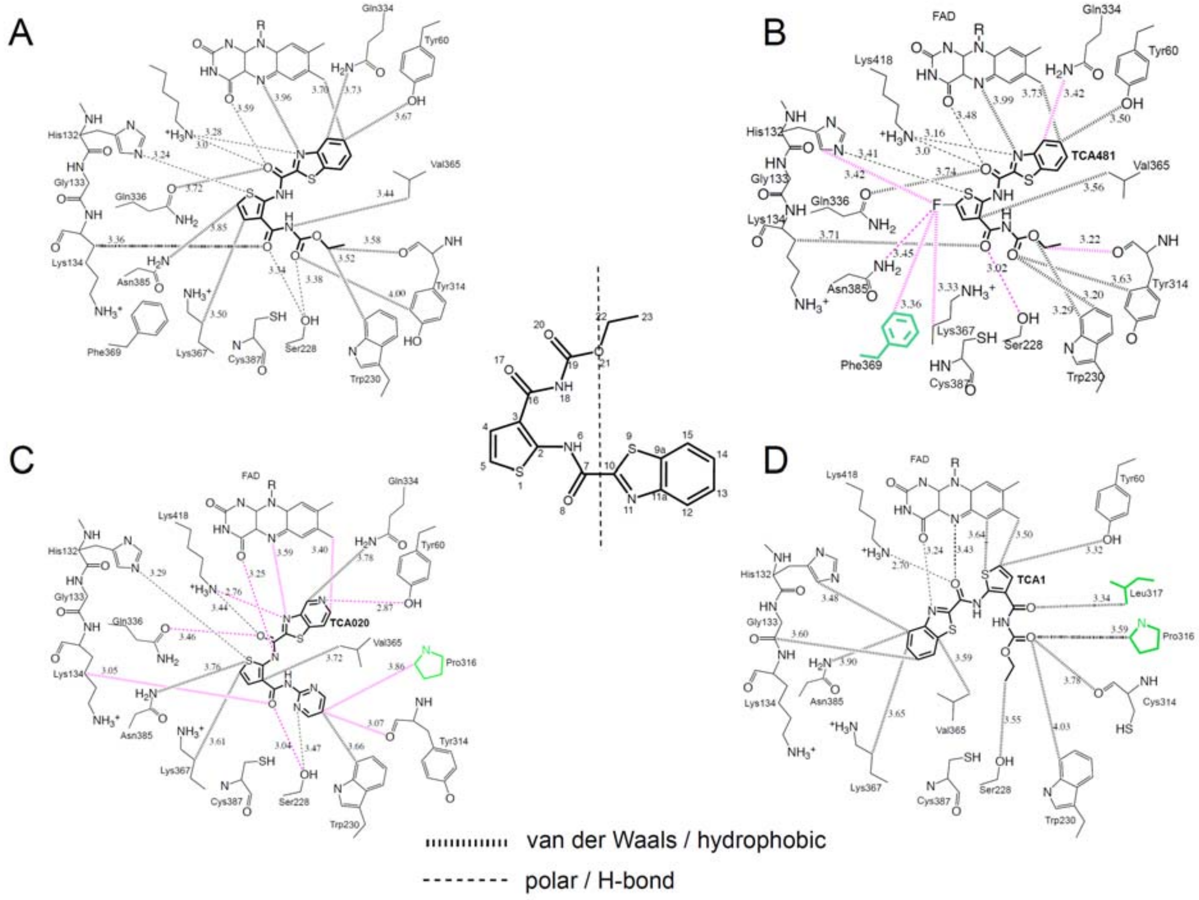

Figure S1 **Schematic diagram of non-covalent interactions of TCA1-derivatives with DprE1.** Distances are indicated in units of Å and, for clarity, only the shortest contacts (within a 4 Å distance cut-off) are included. Non-covalent contacts between DprE1 and (**A**) TCA1 (PDB entry 4KW5), (**B**) TCA481, and (**C**) TCA020. (**D**) Non-covalent contacts of TCA1 when bound to DprE1-Y314C. Central panel: TCA1 with numbered atom positions and indication of the 180º-rotation axis that links the different orientations of the inhibitor in the active site of wt-DprE1 vs DprE1-Y314C.

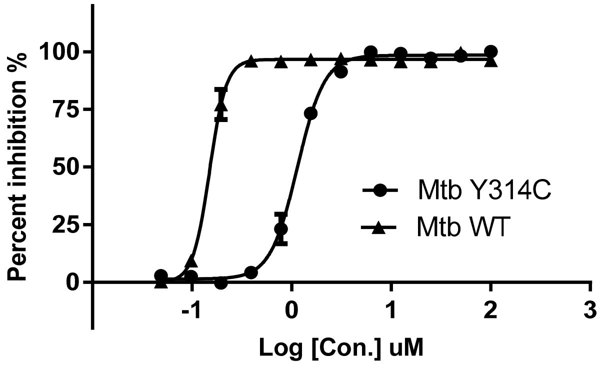

Figure S2. In vitro activity of TCA007 against Mtb wild type (WT) and Mtb Y314C strain.

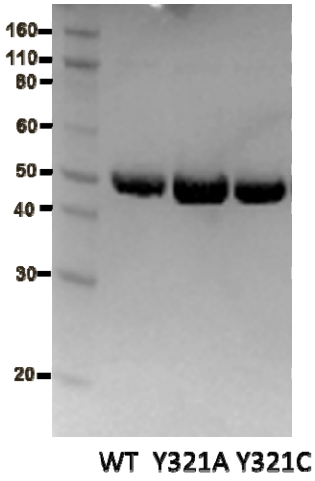

Figure S3. SDS-PAGE analysis of purified *M. smegmatis* DprE1

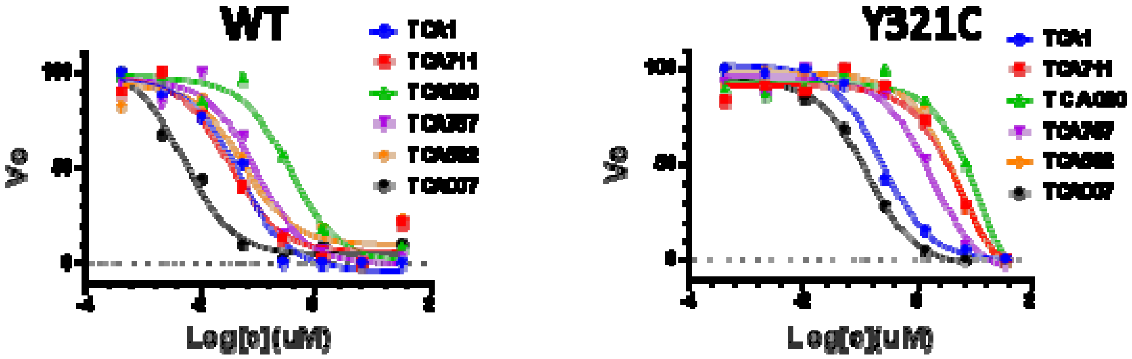

Figure S4. Dose-response curves for TCA1 and 5 analogues against the DprE1 WT and Y321C mutant.

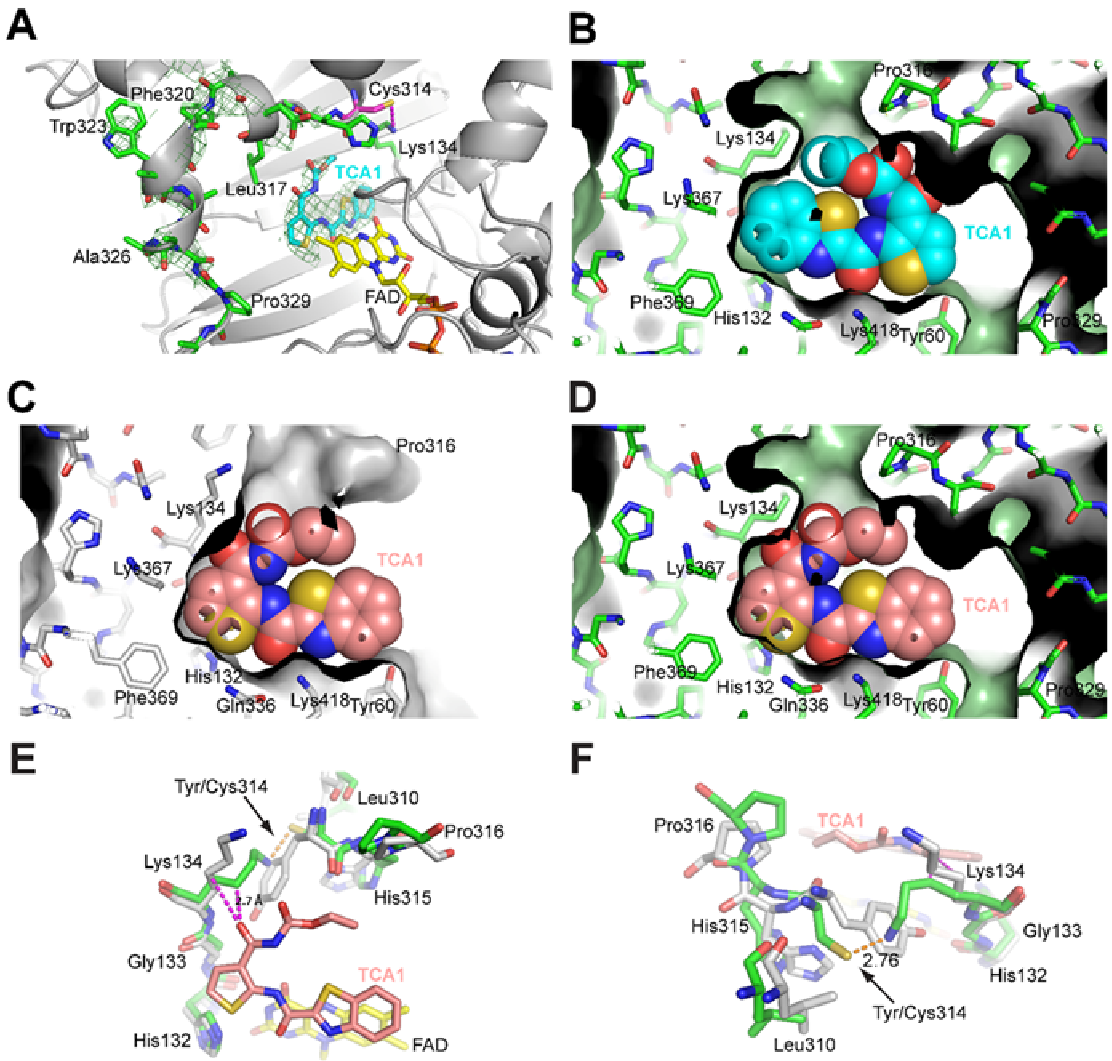

Figure S5. **Altered orientation of TCA1 when bound to DprE1-Y314C**. (**A**) Evidence for the partial ordering of the 315-329 loop in molecule A of DprE1-Y314C bound to TCA1. The difference electron density is shown at a contour level of 3.0 σ. The mutation site (residue 314) is highlighted in magenta, the inhibitor TCA1 shown in cyan. (**B**) Molecular surface of DprE1-Y314C (green) and van der Waals representation of TCA1, illustrating shape complementarity between TCA1 and the active site of DprE1-Y314C. (**C**) For comparison, molecular surface of wild-type DprE1 bound to TCA1 (PDB entry 4KW5). (**D**) Illustration of the steric hindrance between TCA1 and DprE1-Y314C when the inhibitor is oriented as in the wild-type complex. View resulting from superimposing the backbones of wt-DprE1 and DprE1-Y314C. (**E**, **F**) Illustration of structural changes resulting from the resistance-conferring substitution of tyrosine by cysteine at position 314. Sticks are coloured in green and grey for DprE1-Y314C and wt-DprE1, respectively. The steric clash with Lys134 induced by the Y314C substitution is illustrated by the dashed distance markers.

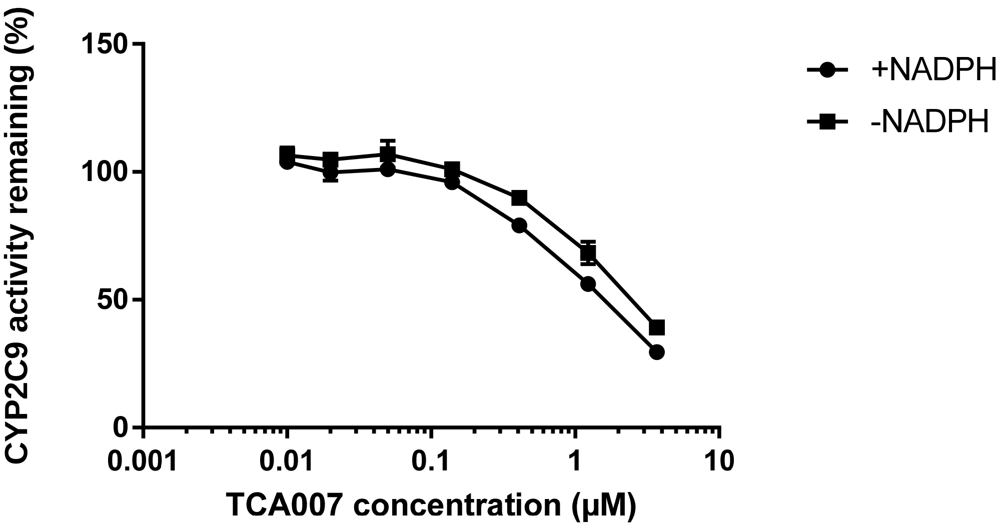

Figure S6. Inhibition of CYP2C9 activity by TCA007 in human liver microsomes with pre-incubation in the presence or absence of NADPH

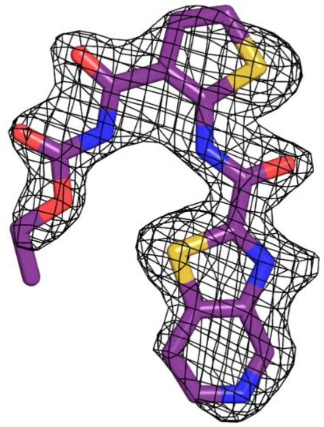

Figure S7. 2mFo-DFc TCA007 omit map contoured at 1 σ around TCA007.

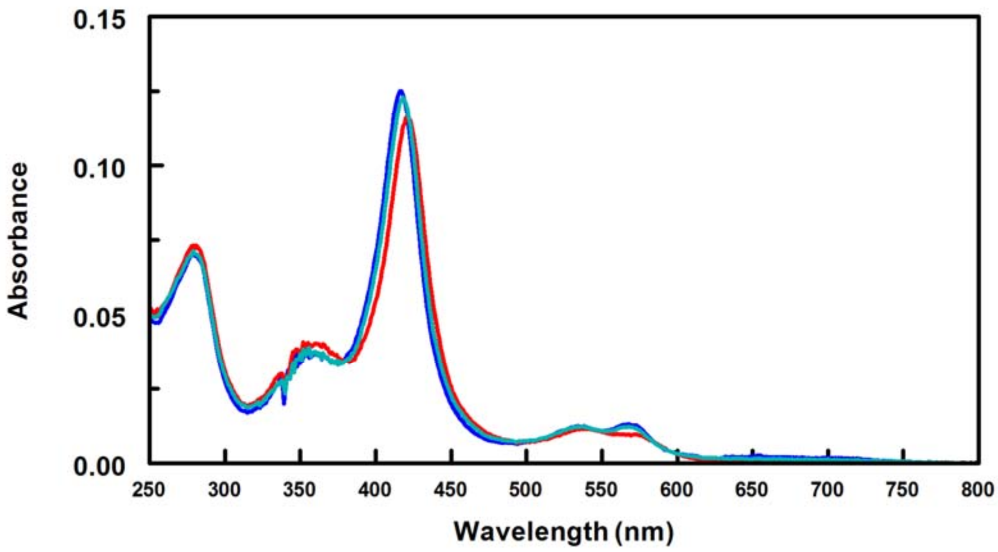

Figure S8. Visible Absorption Spectra of 2C9dH in the presence or absence of miconazole, and the effects TCA007 on the spectral changes elicited by miconazole. The visible absorption spectrum of 2C9dH (1.13 µM) in 1 mL of 20 mM KPi, pH 7.4, 500 mM NaCl, 20% gylcerol, 0.5 mM EDTA buffer is shown by the dark blue line. Addition of miconazole 0.5 µL to the sample and reference cuvettes at final concentration of 2 µM leads to a shift of the Soret band of 2C9dH from 417 nm to 421 nm and diminished intensity of the Q-band at 569 nm (red line) indicative of coordination of the azole nitrogen to the heme iron of 2C9dH. Subsequent addition of TCA007 (4.2 µL in methanol) to a final concentration of 60 µM in each cuvette reverts the spectrum (cyan) to that seen for 2C9dH in the absence of a ligand. This suggests that when TCA007 binds it displaces miconazole without forming a bond between its pyridyl nitrogen and the heme iron consistent with binding seen in the X-ray crystal structure of the complex.

Tables

Table S1. Data and Model Refinement Statistics for each complex structure

X-ray diffraction data and structure refinement.

**X-ray diffraction data**			
**PDB entry**	5OEL	5OEQ	5OEP
Crystal	DprE1(Y314C):TCA1	DprE1:TCA020	DprE1: TCA481
Beamline	Diamond, I03	Diamond, I02	Diamond, I02
Wavelength (Å)	0.9763	0.9795	0.9795
Space group	$P2_1$	$P2_1$	$P2_1$
Cell parameters a,b,c (Å)	77.6, 83.8, 80.6, β=103.2º	78.1, 84.3, 81.2 β=103.7	78.3, 84.5, 81.0 β=103.5
Molecules per asymmetric unit	2	2	2
Resolution (Å)	83.7 – 2.20	57.6 – 2.25	57.6 – 2.35
High resolution shell (Å)	2.26 – 2.20	2.31 – 2.25	2.41 – 2.35
Rmerge (%)	5.0 (57.9)	7.3 (76.9)	3.7 (63.3)
Total observations	188537	239225	143401
unique reflections	50740	48308	42105
I/σ(I)[1)]	13.1 (2.4)	11.2 (2.9)	17.0 (1.9)
Completeness (%)[1)]	99.2 (98.9)	99.3 (99.1)	98.3 (98.1)
Multiplicity[1)]	3.7 (3.8)	5.0 (5.1)	3.4 (3.6)
$CC_{1/2}$[2)]	0.999 (0.811)	0.997 (0.798)	0.998 (0.814)
**Refinement**			
Resolution range	83.7 – 2.2	57.6 - 2.25	57.6 – 2.35
Unique reflections	48146	45918	39956
R_{cryst} (%)	19.6	18.6	19.6
R_{free} (%)	22.3	21.5	23.3
Number of non-hydogen atoms	6727	6524	6569
Protein + FAD	6636	6367	6452
Inhibitor	50	26	52
Solvent	41	126	65
RMSD bonds (Å), angles (º)	0.012, 1.59	0.012, 1.63	0.013, 1.43
**B-factors**			
Wilson (Å^2)	50.3	57.9	61.0
Average (Å^2)	47.0	60.4	64.2
Protein + FAD (Å^2)	47.6	60.6	64.8
Ligand (Å^2)	74.3	63.5	92.3
Solvent (Å^2)	36.9	47.3	47.8
RMSD B-factors(Å^2)	1.42	1.97	2.17
**Ramachandran Plot**[3)]			
Favoured region (%)	97.44	97.71	97.7
Disallowed (%)	0.12	0.12	0.12
Molprobity score (percentile) / comparator group	1.65 (97th) / N=10167, 2.20Å ± 0.25Å	1.76 (95th) / N=11467, 2.25Å ± 0.25Å	1.69 (98th) / N=9377, 2.35 Å ± 0.25 Å

[1)] Values in parentheses refer to high resolution shell. [2)] Correlation between random half-sets of data.
[3)]Calculated using MolProbity (REF).

Table S2. Steady-state kinetics of M.smegmatis DprE1 WT and mutants

	Km (mM)	Ki (uM)
DprE1 WT	6.4	0.15
DprE1 Y321A	3.3	0.17
DprE1 Y321C	2.4	0.56

Table S3. Data and Model Refinement Statistics for P450 2C9 TCA007 Complex

Data Refinement		Model Refinement	PDB: 5W0C
Space Group	H3	Resolution (Å)	38.54-2.00 (2.06-2.00)
Cell Dimensions		Unique Reflections	35497 (2695)
a, b, c (Å)	91.39, 91.39, 169.23	Test Set (%)	4.84
α, β, γ (º)	90, 90, 120	R work (%)	18.15 (26.80)
Resolution (Å)	38.54-2.00 (2.05-2.00)*	R test (%)	21.53 (30.16)
Unique Reflections	35507 (2598)	Heavy Atoms	3997
R merge	0.045 (0.436)	RMSD Bond Lengths	0.008 Å
CC1/2 (%)	99.9 (88.4)	RMSD Bond Angles	0.881º
Mean ((I)/sd(I))	21.6 (4.1)	Ramachandran Plot	Molprobity
Completeness (%)	99.7 (97.9)	Favored (%)	96.8
Multiplicity	5.8 (5.7)	Allowed (%)	3.0
Wilson B Factor (Å^2)	31.16	Outliers (%)	0.2
Protein Chains in ASU	1	Mean B Factors (Å^2)	
		Protein	35.6
		Heme	19.9
		TCA007	44.8
		Solvent	37.8

*Values for in parentheses are for the high resolution shell.

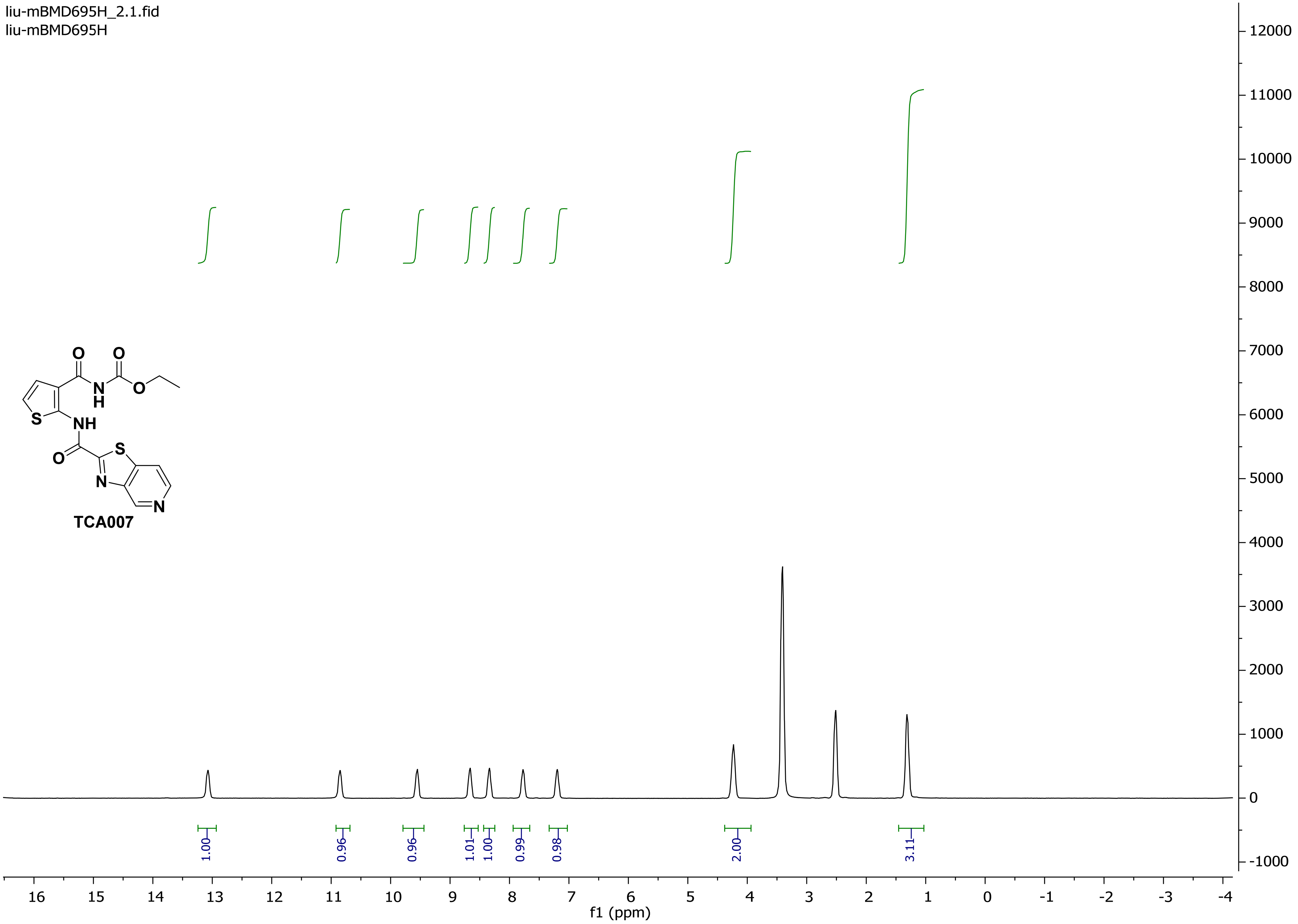
liu-mBMD695H_2.1.fid
liu-mBMD695H
TCA007
1.00
0.96
0.96
1.01
1.00
0.99
0.98
2.00
3.11
f1 (ppm)

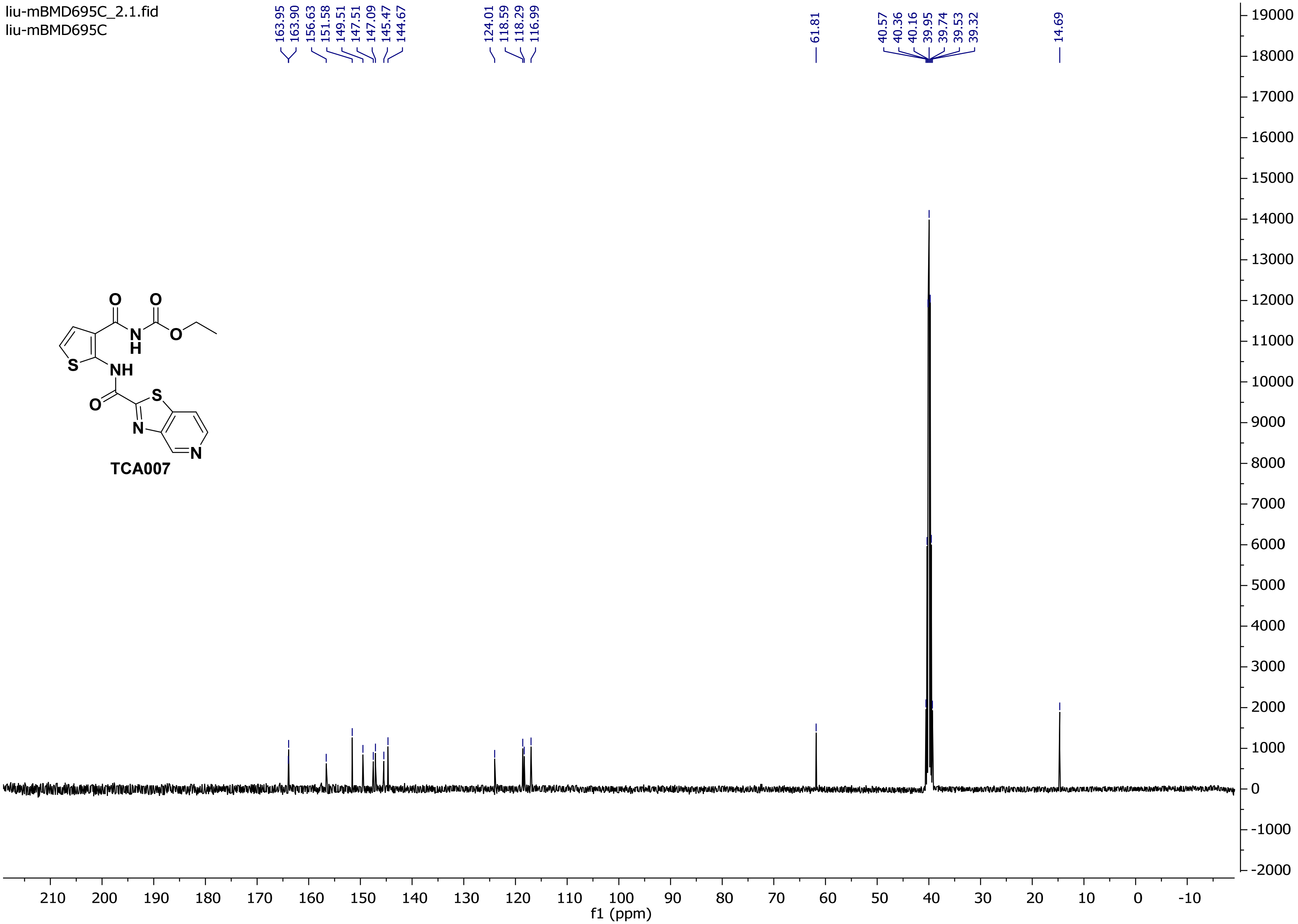
liu-mBMD695C_2.1.fid
liu-mBMD695C
163.95
163.90
156.63
151.58
149.51
147.51
147.09
145.47
144.67
124.01
118.59
118.29
116.99
61.81
40.57
40.36
40.16
39.95
39.74
39.53
39.32
14.69
TCA007
f1 (ppm)

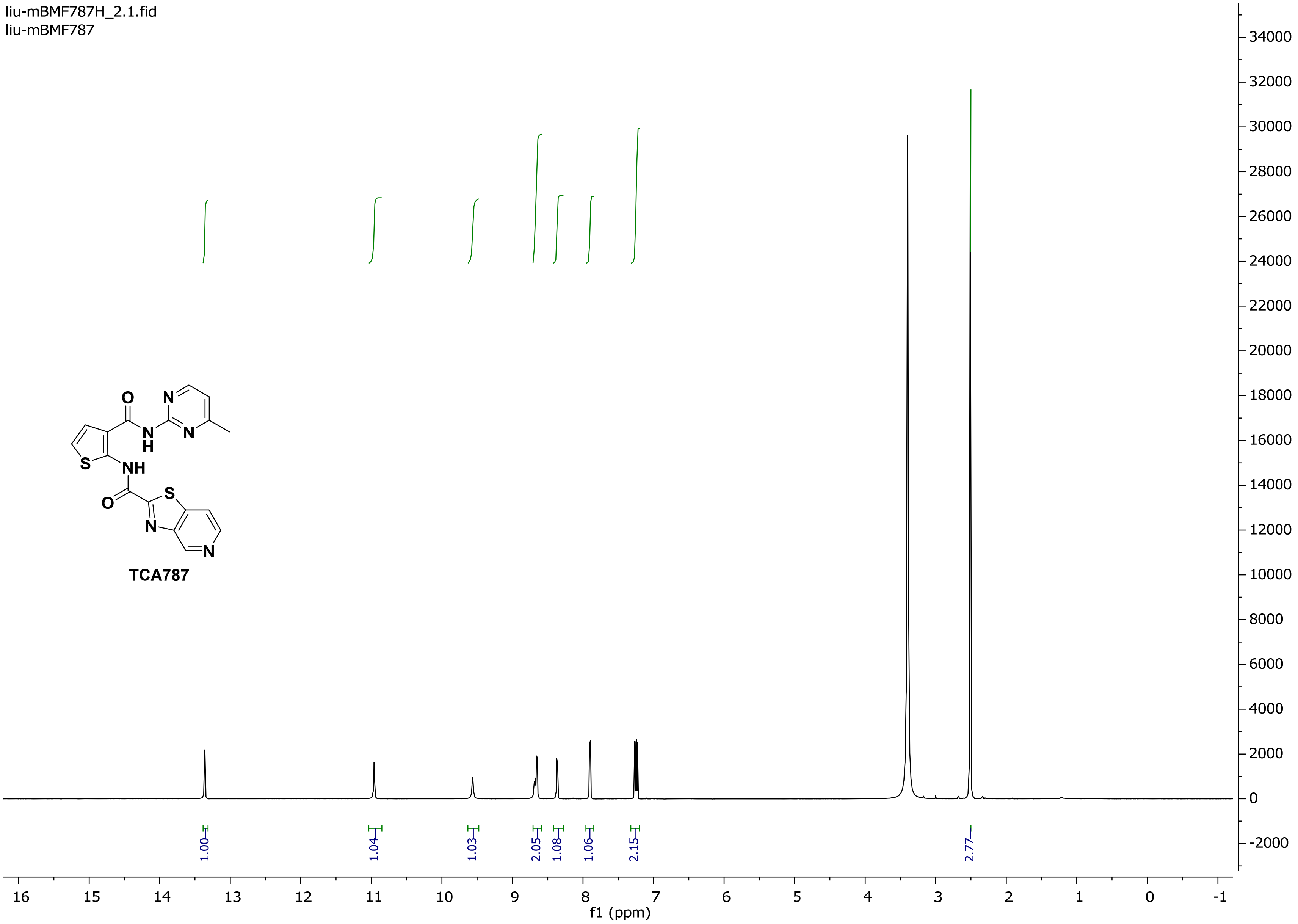
liu-mBMF787H_2.1.fid
liu-mBMF787
TCA787
1.00
1.04
1.03
2.05
1.08
1.06
2.15
2.77
f1 (ppm)

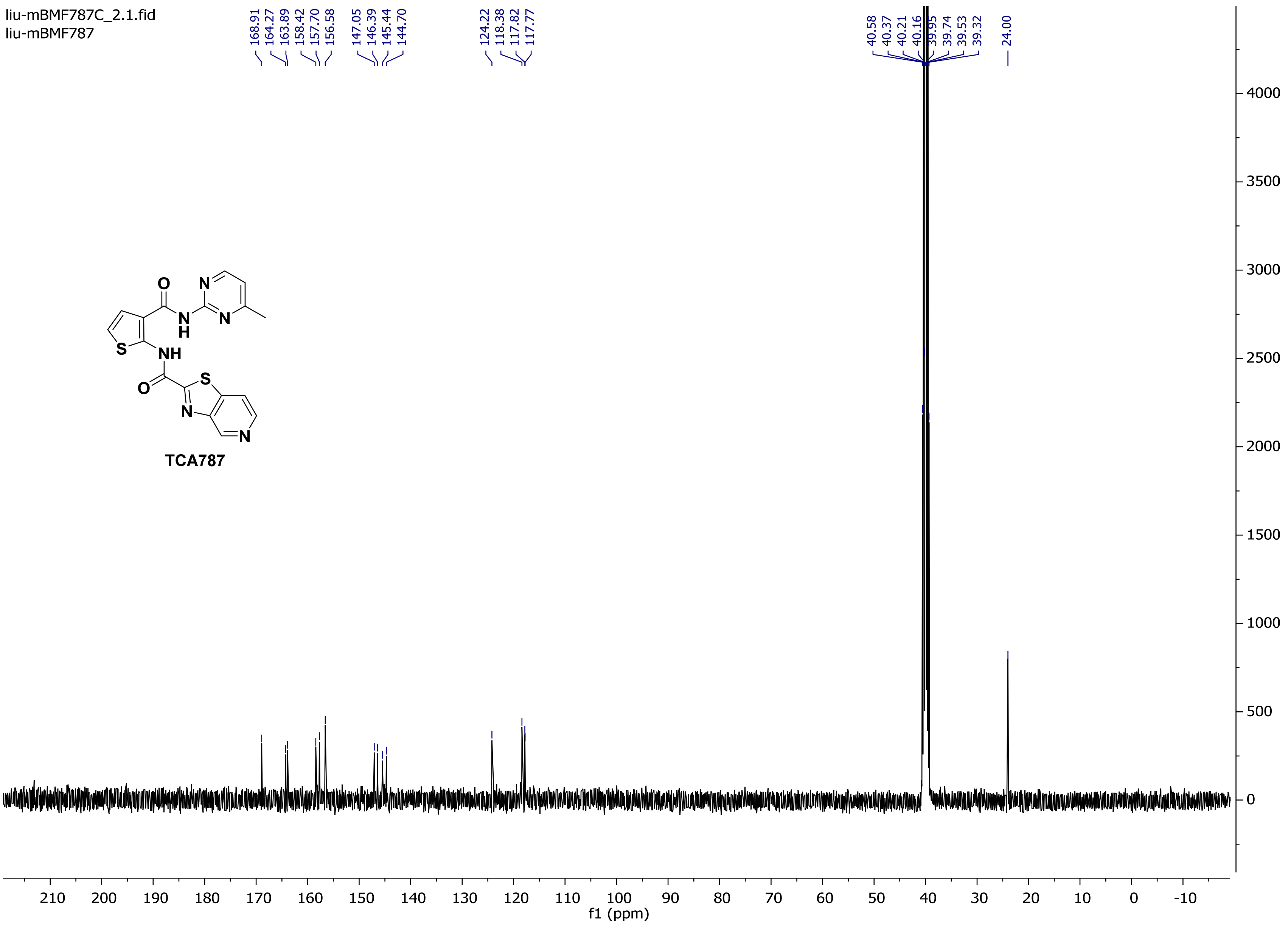
liu-mBMF787C_2.1.fid
liu-mBMF787
168.91
164.27
163.89
158.42
157.70
156.58
147.05
146.39
145.44
144.70
124.22
118.38
117.82
117.77
40.58
40.37
40.21
40.16
39.95
39.74
39.53
39.32
24.00
TCA787
f1 (ppm)

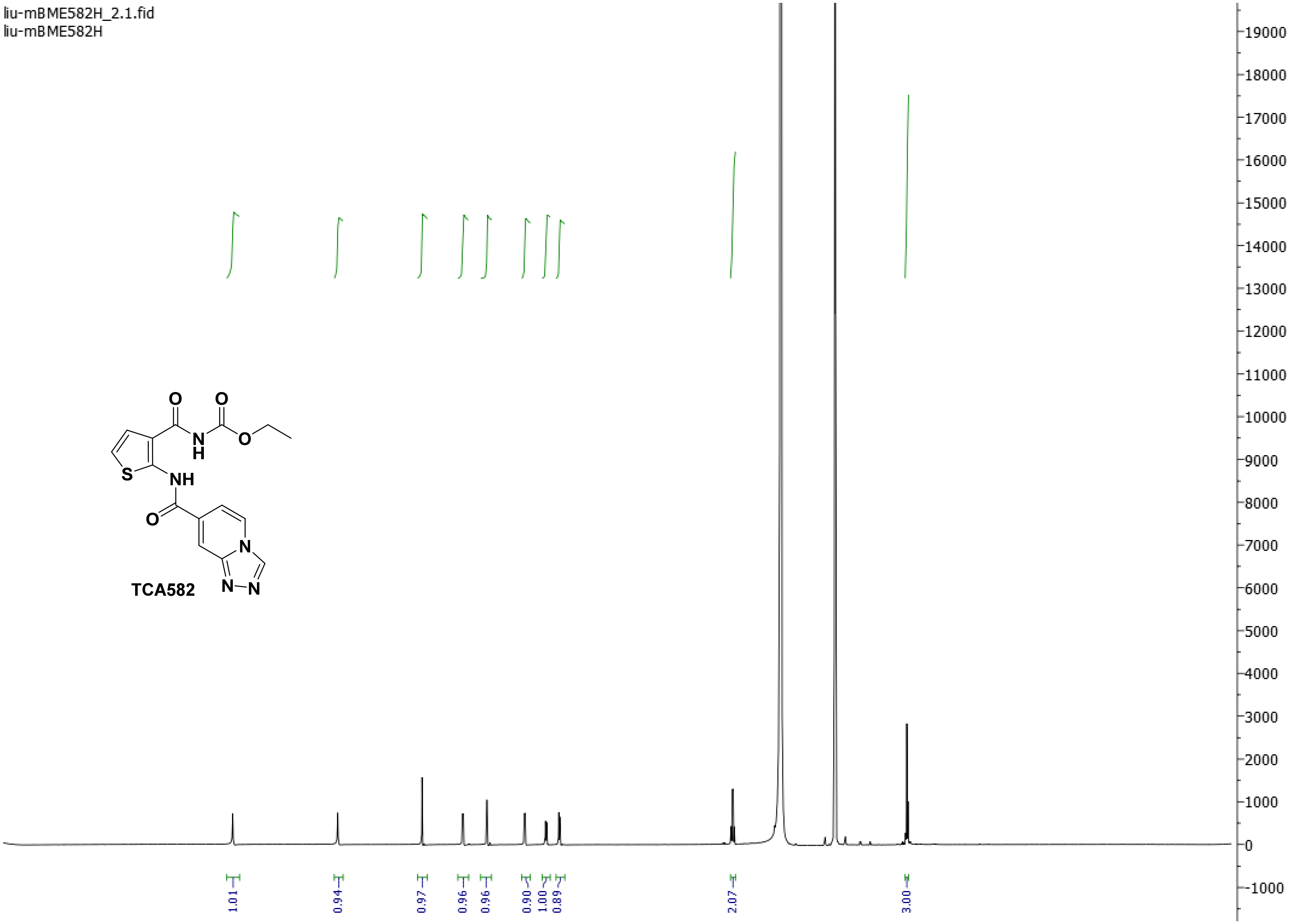
liu-mBME582H_2.1.fid
liu-mBME582H
TCA582
1.01
0.94
0.97
0.96
0.96
0.90
1.00
0.89
2.07
3.00
19000
18000
17000
16000
15000
14000
13000
12000
11000
10000
9000
8000
7000
6000
5000
4000
3000
2000
1000
0
-1000

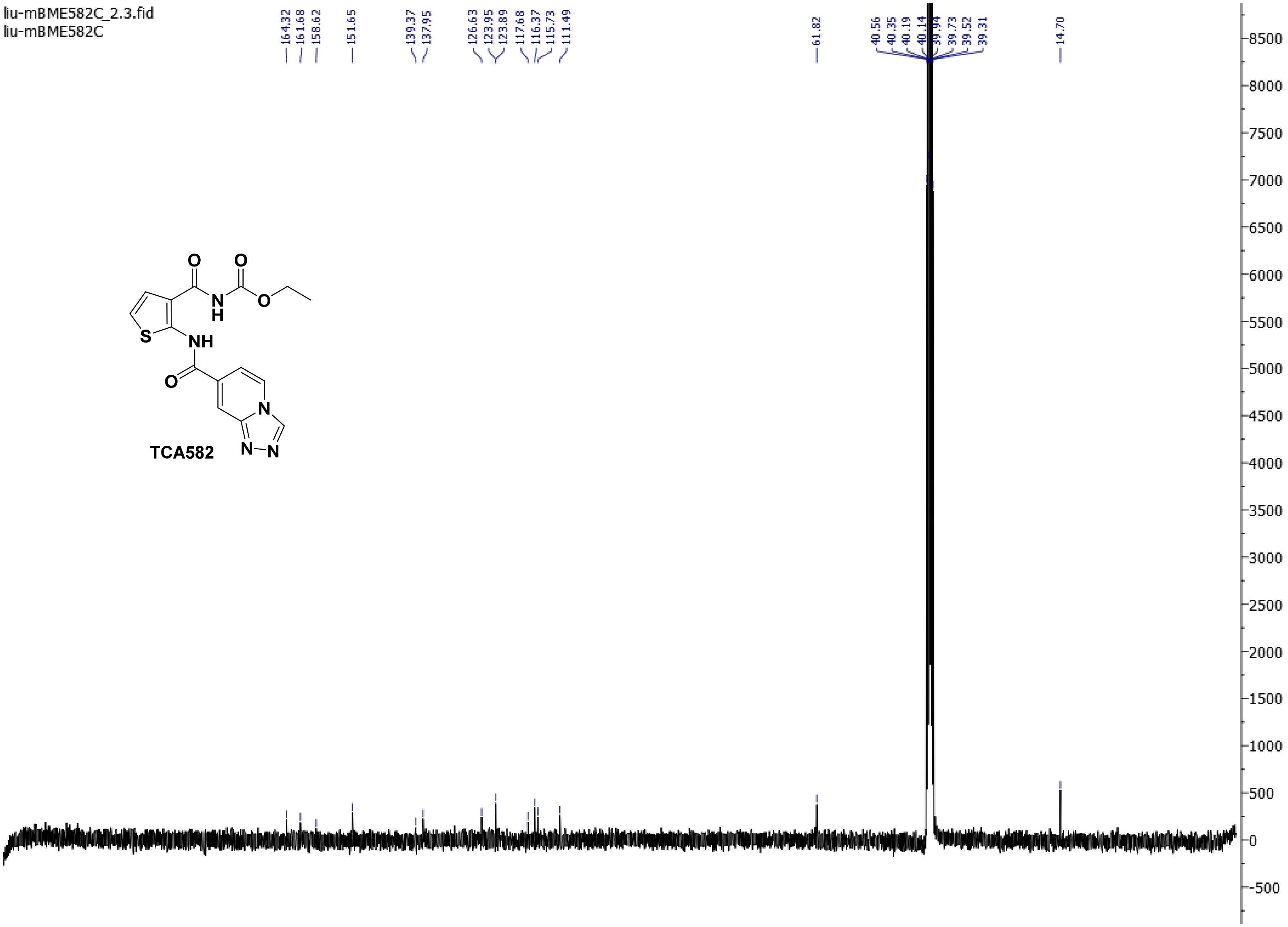
liu-mBME582C_2.3.fid
liu-mBME582C
164.32
161.68
158.62
151.65
139.37
137.95
126.63
123.95
123.89
117.68
116.37
115.73
111.49
61.82
40.56
40.35
40.19
40.14
39.94
39.73
39.52
39.31
14.70
8500
8000
7500
7000
6500
6000
5500
5000
4500
4000
3500
3000
2500
2000
1500
1000
500
0
-500
TCA582

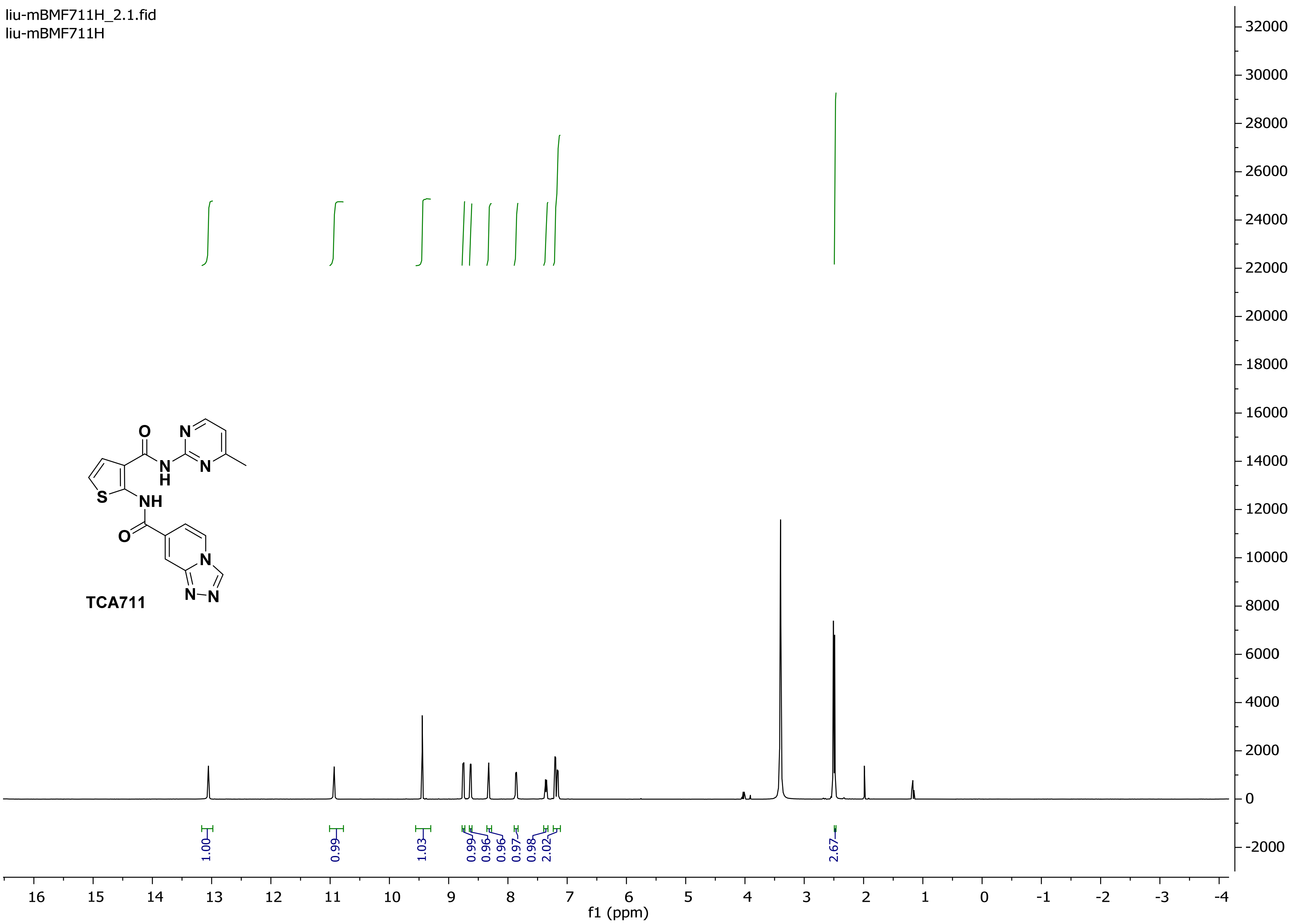
liu-mBMF711H_2.1.fid
liu-mBMF711H
TCA711
f1 (ppm)

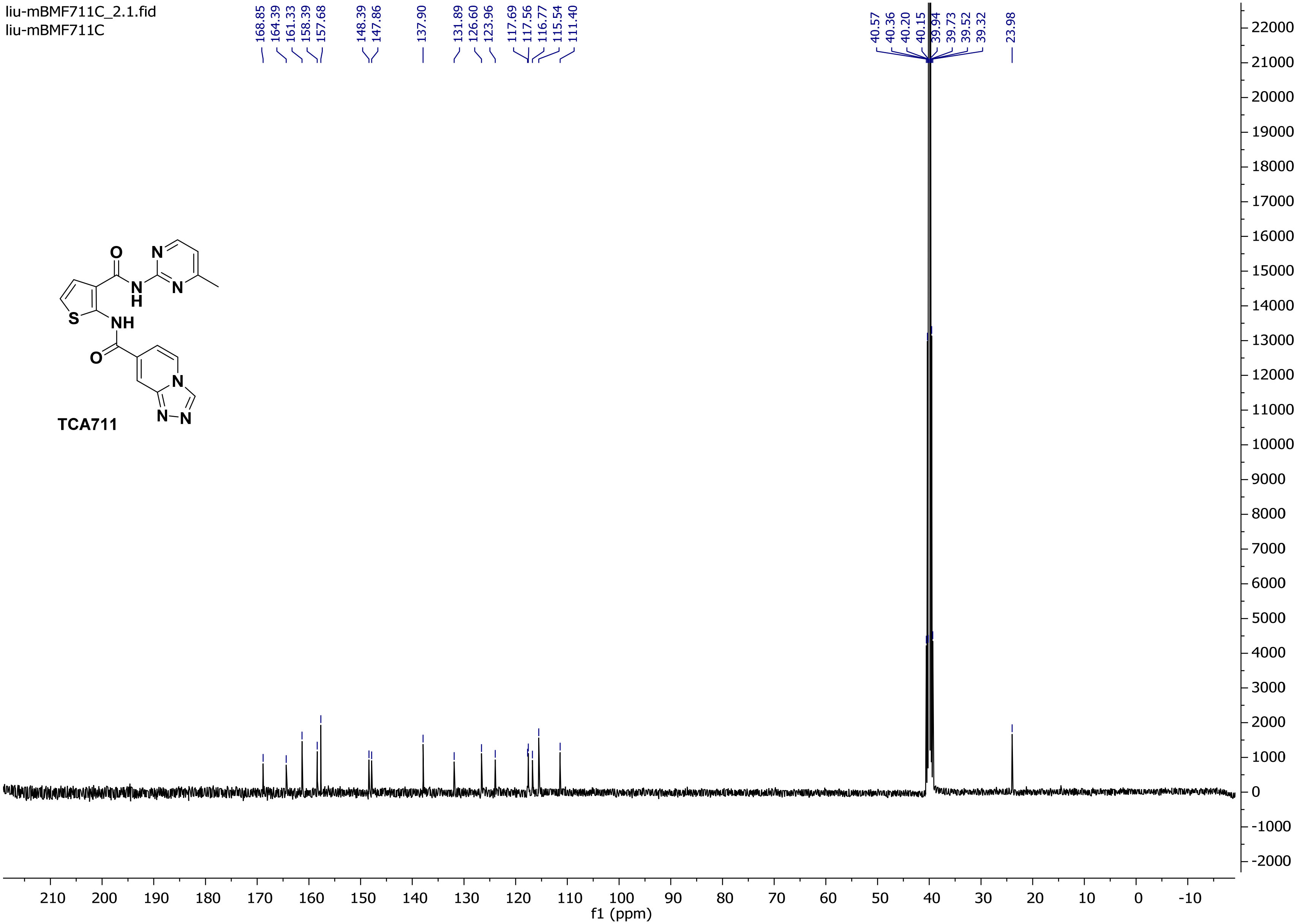
liu-mBMF711C_2.1.fid
liu-mBMF711C
168.85
164.39
161.33
158.39
157.68
148.39
147.86
137.90
131.89
126.60
123.96
117.69
117.56
116.77
115.54
111.40
40.57
40.36
40.20
40.15
39.94
39.73
39.52
39.32
23.98
TCA711
f1 (ppm)

References in the main text with complete author names

[1] B. A. Wolucka, *FEBS J.* **2008**, *275*, 2691.

[2] G. S. Kolly, F. Boldrin, C. Sala, N. Dhar, R. C. Hartkoorn, M. Ventura, A. Serafini, J. D. McKinney, R. Manganelli, S. T. Cole, *Mol. Microbiol.* **2014**, *92*, 194.

[3] a) V. Makarov, G. Manina, K. Mikusova, U. Mollmann, O. Ryabova, B. Saint-Joanis, N. Dhar, M. R. Pasca, S. Buroni, A. P. Lucarelli, A. Milano, E. De Rossi, M. Belanova, A. Bobovska, P. Dianiskova, J. Kordulakova, C. Sala, E. Fullam, P. Schneider, J. D. McKinney, P. Brodin, T. Christophe, S. Waddell, P. Butcher, J. Albrethsen, I. Rosenkrands, R. Brosch, V. Nandi, S. Bharath, S. Gaonkar, R. K. Shandil, V. Balasubramanian, T. Balganesh, S. Tyagi, J. Grosset, G. Riccardi, S. T. Cole, *Science* **2009**, *324*, 801; b) M. R.-G. Claudia Trefzer, Marlon J. Hinner, Patricia Schneider, Vadim Makarov, Stewart T. Cole, and Kai Johnsson, *J. Am. Chem. Soc.* **2010**, *132*, 13663; c) J. Neres, F. Pojer, E. Molteni, L. R. Chiarelli, N. Dhar, S. Boy-Rottger, S. Buroni, E. Fullam, G. Degiacomi, A. P. Lucarelli, R. J. Read, G. Zanoni, D. E. Edmondson, E. De Rossi, M. R. Pasca, J. D. McKinney, P. J. Dyson, G. Riccardi, A. Mattevi, S. T. Cole, C. Binda, *Sci. Transl. Med.* **2012**, *4*, 150ra121; d) C. Trefzer, H. Skovierova, S. Buroni, A. Bobovska, S. Nenci, E. Molteni, F. Pojer, M. R. Pasca, V. Makarov, S. T. Cole, G. Riccardi, K. Mikusova, K. Johnsson, *J. Am. Chem. Soc.* **2012**, *134*, 912; e) R. Tiwari, G. C. Moraski, V. Krchnak, P. A. Miller, M. Colon-Martinez, E. Herrero, A. G. Oliver, M. J. Miller, *J. Am. Chem. Soc.* **2013**, *135*, 3539.

[4] a) M. Naik, V. Humnabadkar, S. J. Tantry, M. Panda, A. Narayan, S. Guptha, V. Panduga, P. Manjrekar, L. K. Jena, K. Koushik, G. Shanbhag, S. Jatheendranath, M. R. Manjunatha, G. Gorai, C. Bathula, S. Rudrapatna, V. Achar, S. Sharma, A. Ambady, N. Hegde, J. Mahadevaswamy, P. Kaur, V. K. Sambandamurthy, D. Awasthy, C. Narayan, S. Ravishankar, P. Madhavapeddi, J. Reddy, K. Prabhakar, R. Saralaya, M. Chatterji, J. Whiteaker, B. McLaughlin, L. R. Chiarelli, G. Riccardi, M. R. Pasca, C. Binda, J. Neres, N. Dhar, F. Signorino-Gelo, J. D. McKinney, V. Ramachandran, R. Shandil, R. Tommasi, P. S. Iyer, S. Narayanan, V. Hosagrahara, S. Kavanagh, N. Dinesh, S. R. Ghorpade, *J. Med. Chem.* **2014**, *57*, 5419; b) M. Panda, S. Ramachandran, V. Ramachandran, P. S. Shirude, V. Humnabadkar, K. Nagalapur, S. Sharma, P. Kaur, S. Guptha, A. Narayan, J. Mahadevaswamy, A. Ambady, N. Hegde, S. S. Rudrapatna, V. P. Hosagrahara, V. K. Sambandamurthy, A. Raichurkar, *J. Med. Chem.* **2014**, *57*, 4761; c) P. S. Shirude, R. K. Shandil, M. R. Manjunatha, C. Sadler, M. Panda, V. Panduga, J. Reddy, R. Saralaya, R. Nanduri, A. Ambady, S. Ravishankar, V. K. Sambandamurthy, V. Humnabadkar, L. K. Jena, R. S. Suresh, A. Srivastava, K. R. Prabhakar, J. Whiteaker, R. E. McLaughlin, S. Sharma, C. B. Cooper, K. Mdluli, S. Butler, P. S. Iyer, S. Narayanan, M. Chatterji, *J. Med. Chem.* **2014**, *57*, 5728; d) S. M. Batt, M. Cacho Izquierdo, J. Castro Pichel, C. J. Stubbs, L. Vela-Glez Del Peral, E. Perez-Herran, N. Dhar, B. Mouzon, M. Rees, J. P. Hutchinson, R. J. Young, J. D. McKinney, D. Barros Aguirre, L. Ballell, G. S. Besra, A. Argyrou, *ACS Infect. Dis.* **2015**, *1*, 615; e) J. Neres, R. C. Hartkoorn, L. R. Chiarelli, R. Gadupudi, M. R. Pasca, G. Mori, A. Venturelli, S. Savina, V. Makarov, G. S. Kolly, E. Molteni, C. Binda, N. Dhar, S. Ferrari, P. Brodin, V. Delorme, V. Landry, A. L. de Jesus Lopes Ribeiro, D. Farina, P. Saxena, F. Pojer, A. Carta, R. Luciani, A. Porta, G. Zanoni, E. De Rossi, M. P. Costi, G. Riccardi, S. T. Cole, *ACS Chem. Biol.* **2015**, *10*, 705.

[5] M. Brecik, I. Centarova, R. Mukherjee, G. S. Kolly, S. Huszar, A. Bobovska, E. Kilacskova, V. Mokosova, Z. Svetlikova, M. Sarkan, J. Neres, J. Kordulakova, S. T. Cole, K. Mikusova, *ACS Chem. Biol.* **2015**, *10*, 1631.

[6] F. Wang, D. Sambandan, R. Halder, J. Wang, S. M. Batt, B. Weinrick, I. Ahmad, P. Yang, Y. Zhang, J. Kim, M. Hassani, S. Huszar, C. Trefzer, Z. Ma, T. Kaneko, K. E. Mdluli, S. Franzblau, A. K. Chatterjee, K. Johnsson, K. Mikusova, G. S. Besra, K. Futterer, S. H. Robbins, S. W. Barnes, J. R. Walker, W. R. Jacobs, Jr., P. G. Schultz, *Proc. Natl. Acad. Sci. U.S.A* **2013**, *110*, E2510.

[7] D. O. Nettleton, H. J. Einolf, *Curr. Top. Med. Chem.* **2011**, *11*, 382.

[8] A. M. Upton, S. Cho, T. J. Yang, Y. Kim, Y. Wang, Y. Lu, B. Wang, J. Xu, K. Mdluli, Z. Ma, S. G. Franzblau, *Antimicrob. Agents. Chemother.* **2015**, *59*, 136.

[9] P. M. Dansette, G. Bertho, D. Mansuy, *Biochem. Biophys. Res. Commun.* **2005**, *338*, 450.

[10] M. P. Lopez-Garcia, P. M. Dansette, D. Mansuy, *Biochemistry* **1994**, *33*, 166.

[11] a) M. R. Wester, J. K. Yano, G. A. Schoch, C. Yang, K. J. Griffin, C. D. Stout, E. F. Johnson, *J. Biol. Chem.* **2004**, *279*, 35630; b) P. A. Williams, J. Cosme, A. Ward, H. C. Angove, D. Matak Vinkovic, H. Jhoti, *Nature* **2003**, *424*, 464.

[12] M. Ridderstrom, C. Masimirembwa, S. Trump-Kallmeyer, M. Ahlefelt, C. Otter, T. B. Andersson, *Biochem. Biophys. Res. Commun.* **2000**, *270*, 983.

[13] L. J. Dickmann, C. W. Locuson, J. P. Jones, A. E. Rettie, *Mol. Pharmacol.* **2004**, *65*, 842.
